# Cellular mechanisms underlying carry-over effects after magnetic stimulation

**DOI:** 10.1038/s41598-024-55915-8

**Published:** 2024-03-02

**Authors:** Hui Ye, Maria Dima, Vincent Hall, Jenna Hendee

**Affiliations:** Department of Biology, Loyola University Chicago, Quinlan Life Sciences Education and Research Center, 1032 W. Sheridan Rd., Chicago, IL 60660 USA

**Keywords:** Magnetic stimulation, Miniature coil, Inhibition, *Aplysia californica*, Neuron, Carry-over effects, NEURON modeling, Ion channel, Electrophysiology, Cellular neuroscience, Computational neuroscience, Biophysics

## Abstract

Magnetic fields are widely used for neuromodulation in clinical settings. The intended effect of magnetic stimulation is that neural activity resumes its pre-stimulation state right after stimulation. Many theoretical and experimental works have focused on the cellular and molecular basis of the acute neural response to magnetic field. However, effects of magnetic stimulation can still last after the termination of the magnetic stimulation (named “carry-over effects”), which could generate profound effects to the outcome of the stimulation. However, the cellular and molecular mechanisms of carry-over effects are largely unknown, which renders the neural modulation practice using magnetic stimulation unpredictable. Here, we investigated carry-over effects at the cellular level, using the combination of micro-magnetic stimulation (µMS), electrophysiology, and computation modeling. We found that high frequency magnetic stimulation could lead to immediate neural inhibition in ganglion neurons from *Aplysia californica*, as well as persistent, carry-over inhibition after withdrawing the magnetic stimulus. Carry-over effects were found in the neurons that fired action potentials under a variety of conditions. The carry-over effects were also observed in the neurons when the magnetic field was applied across the ganglion sheath. The state of the neuron, specifically synaptic input and membrane potential fluctuation, plays a significant role in generating the carry-over effects after magnetic stimulation. To elucidate the cellular mechanisms of such carry-over effects under magnetic stimulation, we simulated a single neuron under magnetic stimulation with multi-compartment modeling. The model successfully replicated the carry-over effects in the neuron, and revealed that the carry-over effect was due to the dysfunction of the ion channel dynamics that were responsible for the initiation and sustaining of membrane excitability. A virtual voltage-clamp experiment revealed a compromised Na conductance and enhanced K conductance post magnetic stimulation, rendering the neurons incapable of generating action potentials and, therefore, leading to the carry over effects. Finally, both simulation and experimental results demonstrated that the carry-over effects could be controlled by disturbing the membrane potential during the post-stimulus inhibition period. Delineating the cellular and ion channel mechanisms underlying carry-over effects could provide insights to the clinical outcomes in brain stimulation using TMS and other modalities. This research incentivizes the development of novel neural engineering or pharmacological approaches to better control the carry-over effects for optimized clinical outcomes.

## Introduction

As a non-invasive method, magnetic fields are widely used for neural stimulation in clinical settings. One well-developed technology is transcranial magnetic stimulation (TMS) of the brain tissue, in which the magnetic field can penetrate the skull without attenuation. By electromagnetic induction, the magnetic pulses then generate an induced electric field inside the neural tissue. Magnetic pulses can also alter the membrane potential in individual neurons, either exciting them^1–3^ or inhibiting them^4^, which is clinically applicable in the treatment of various psychiatric disorders, such as epilepsy and depression.

In order to understand the cellular mechanisms underlying magnetic stimulation, numerous experimental^1,5–7^ and theoretical works^8–11^ have focused on the cellular responses to magnetic stimulation. These works confirmed the causal relationship between the magnetic field and cellular responses, and connected the parameters that define the magnetic stimulation with the instant cellular responses. Recent evidence also demonstrates the impact of the cellular biophysics on the outcome of magnetic stimulation^3,7,12^. Rather than passively responding to the magnetic stimulation, neurons may also affect the outcome of the stimulation with their unique biophysical properties^3^ and respective excitation levels^12^. The instant response of individual cells to the magnetic stimulation is dependent on the dynamic interaction between the magnetic field and the neurons^13,14^.

Aside from the immediate and rapid cellular response to the magnetic stimulation, emerging evidence has shown that the nervous system can be affected by the magnetic stimulation even after the stimulation has been terminated. This phenomenon, termed “carry-over” effects, has been observed in many neuromodulation practices using a magnetic field as the stimulus. For example, repetitive TMS (rTMS) can cause modulation in cortical excitability beyond the duration of the rTMS trains themselves. When applied in sessions of repeated stimulation, TMS can lead to changes in neuronal activity/excitability that can outlast the stimulation itself^15^. A lasting inhibition or facilitation of cortical excitability can be induced by adjusting the rTMS parameters, and can be observed with neurophysiology or imaging techniques^16^.

Previous studies have investigated the underlying mechanisms of post-stimulation effects in magnetic stimulation. Research has focused on changes at the circuit level involving the functional connections, plasticity, and structural changes of the circuit. First, recurrent circuitry has been observed to contribute to carry-over effects. The induced electric field initially depolarizes the membrane of cells in the superficial neural tissue underneath the coil, causing action potentials and a local response in the stimulated region^16^. This local activation propagates to distant cortical and subcortical sites, and can even propagate back from the downstream areas to the primary stimulation site^17^, known as recurrence^18^, which contributes to post-stimulation, or carry-over, effects. Second, carry-over effects relate to the delayed change in the functional connectivity among nodes of the affected network after magnetic stimulation^19,20^. rTMS can change the connections between cortical areas, especially connections between the stimulated cortex and other sites in the brain^21^. When low frequency rTMS was applied to the motor cortex, increased ipsilateral cortico-cortical and interhemispheric coherence in the alpha band were observed. This adapted connectivity resulted in persistent, post-stimulation inhibitory effects^22^. Such observations of altered neural circuits are responsible for an immediate and prolonged reduction of evoked electrophysiology activity^23^. Third, carry-over effects could be due to the post-TMS changes in the balance of excitation and inhibition among functionally connected networks. These excitatory/inhibitory effects have been associated with long-term potentiation (LTP) and depression (LTD) mechanisms, respectively^20,24^. Finally, carry-over effects could be due to long-term structural changes, with contributions to the long-lasting physiological changes observed post rTMS. For example, magnetic stimulation induces a long-lasting increase in glutamatergic synaptic strength, which is accompanied by the structural remodeling of dendritic spines^25^. Systemic work indicates that changes at the large-scale network level could play a significant role in the development of carry-over effects under magnetic stimulation. However, little is known about whether individual neurons, the building blocks of the circuit, could also demonstrate carry-over stimulation effects under magnetic stimulation.

It is not a trivial issue to study the carry-over effects of individual neurons under magnetic stimulation, particularly due to the presence of two technical challenges. First, for a single neuron study, it is essential, yet tedious, to develop a magnetic field that can provide specific, local stimulation at the micro-scale. Second, it is difficult to monitor individual neuronal responses in the midst of the electrically noisy environment. Developed in the last decade, the micromagnetic stimulation (µMS) technology is able to control single neurons due to its excellent resolution in stimulation^1,26^. These microcoils can be miniature in size and covered with soft material to improve biocompatibility. Several recent works have reported the study of individual neurons using intracellular recording^4^ and patch clamp technology^1,27^ under µMS.

We will use neurons from the marine mollusk, *Aplysia californica,* in this study. Since the 1960s, the *Aplysia* neurons have been used for the development of novel neuromodulation technologies, such as high frequency alternating current (HFAC)^28^, infrared^29^, ganglionic surface electrode arrays (GSEAs)^30^, and carbon fiber microelectrodes^31^. The buccal ganglion in *Aplysia* contains large interneurons and motor neurons that control the feeding behavior of the animal. Previous studies have shown that single buccal ganglion neuron activity could be selectively controlled with electric currents in vitro^32^ and in vivo^33^, with an electrode positioned close to the ganglion. The large buccal neurons are easily targeted by the miniature coil. In fact, when a microcoil was positioned close to the soma of the large cell body, it was effective in providing focal stimulation and inhibition of single buccal neurons^4,6,12^.

In this paper, we report lasting inhibitory effects after high frequency magnetic stimulation (i.e., carry-over effects), and investigate the underlying cellular and ion channel mechanisms. Delineating these mechanisms is essential for the control of carry-over effects, and further development of the magnetic stimulation technology for optimal neural control with magnetic stimulation.

## Results

**Figure 1:**
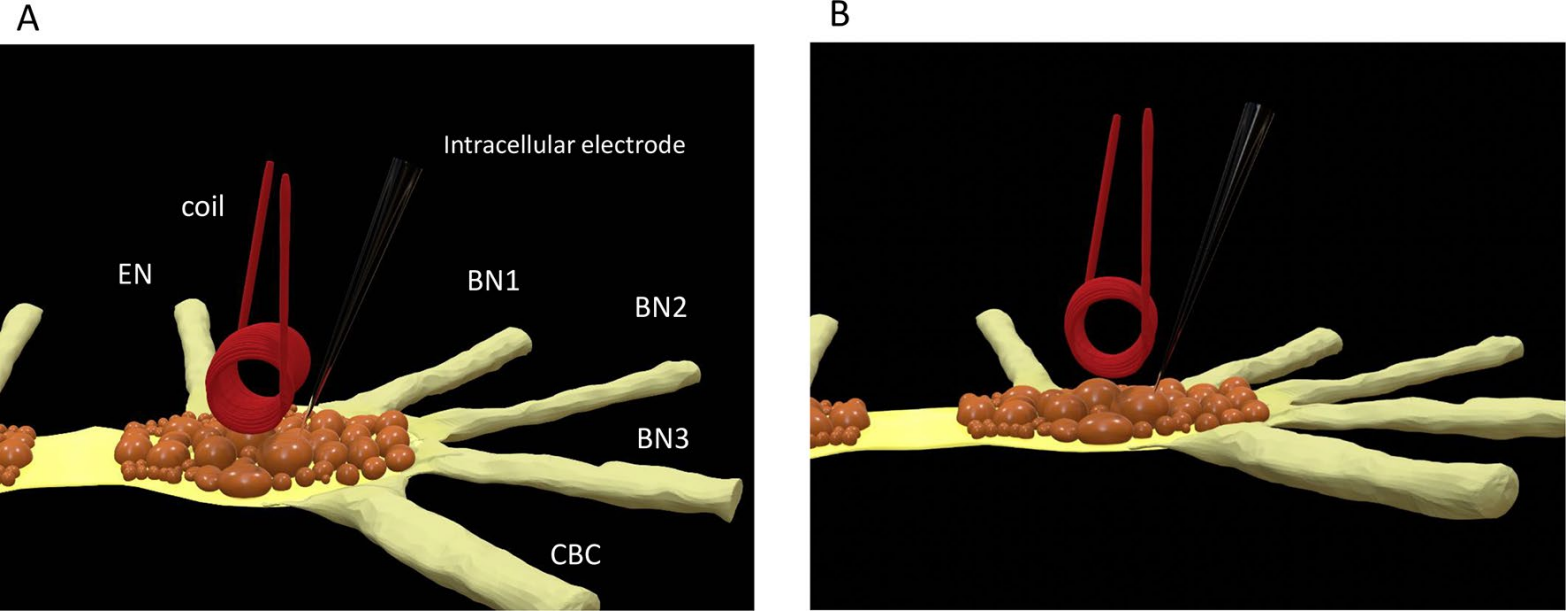
Position of the miniature coil for magnetic stimulation of the neurons in the buccal ganglion from *Aplysia californica*. The schematic drawing indicates the locations of identified large neurons, several nerves attached to the buccal ganglion, the orientation of the coil above the ganglion, and a sharp glass electrode for intracellular recording from a magnetically stimulated neuron. (**A**) Top view; (**B**) side view. EN: esophageal nerve; BN1: buccal nerve I; BN2: buccal nerve II; BN3: buccal nerve III; CBC: cerebro-buccal connection.

**Figure 2:**
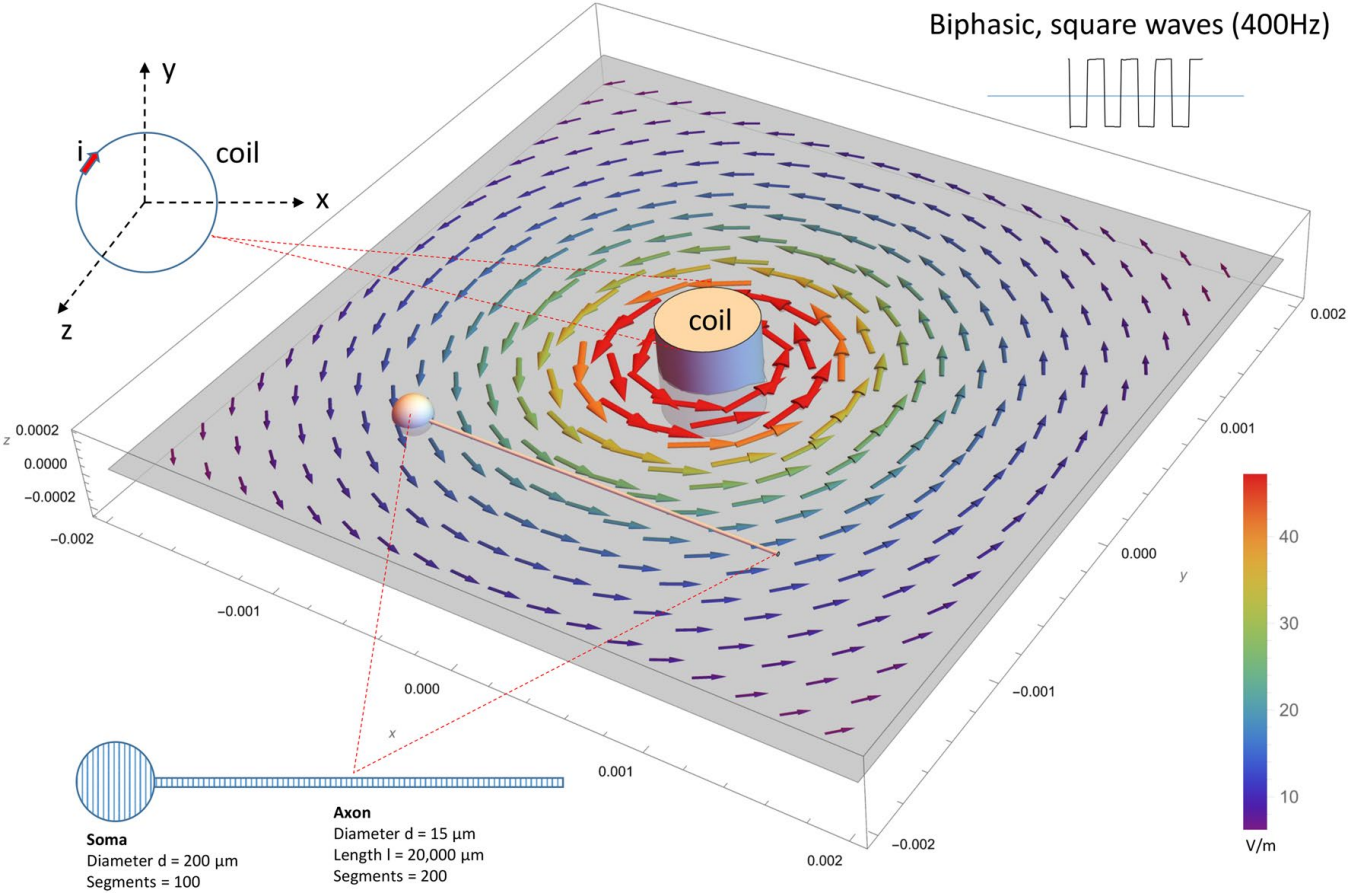
NEURON model of an *Aplysia* buccal neuron stimulated by a miniature magnetic coil. The modeled neuron contained a spherical soma and a cylindrical axon and was divided into many compartments. Each compartment of the model contained H–H type ion channels^4^. High frequency current delivered into the coil generated a biphasic electric field around the neuron. Distribution of the induced electric field around the modeled neuron was illustrated.

In this paper, we used several different protocols to elicit activity in individual neurons in vitro in the buccal ganglia of *Aplysia californica.* We then applied high frequency magnetic stimulation to these neurons, using a submillimeter magnetic coil (Fig. 1). We documented the inhibitory effects during the magnetic stimulation. For the first time, we reported the carry-over, post-stimulation inhibition of individual neurons after magnetic stimulation. We then explored the ion channel mechanisms underlying carry-over inhibition using a multi-compartment NEURON model (Fig. 2). Finally, we introduced and tested strategies to control the carry-over inhibition of the single neurons.

### Magnetic stimulation generated carry-over, post-stimulation inhibition in neurons with spontaneous activity

To monitor the behavior of individual neurons under high frequency magnetic stimulation, we de-sheathed the buccal ganglion from *Aplysia californica* and performed intracellular recordings from the buccal neurons. Activities of these neurons are responsible for the feeding behavior in *Aplysia californica*^34–36^. When the sharp electrode was inserted into the soma, it normally recorded spontaneous activity, which usually lasted for 10–20 min, until the neuron became quiescent again. We tested the capability of the magnetic stimulation on these spontaneous action potentials. We positioned a miniature coil on top of the buccal ganglion (Fig. 1), and delivered high frequency pulses into the coil to provide focal neural stimulation.

Previously, we have found that stimulation with a 400 Hz magnetic field could inhibit the buccal ganglion neurons^4^. Furthermore, we reported that neurons could demonstrate different sensitivities to the magnetic field. The neurons firing at a higher frequency were difficult to inhibit with the high frequency magnetic field, and neurons firing at a lower frequency were easier to inhibit, a phenomenon called state-dependent magnetic inhibition^12^.

**Figure 3:**
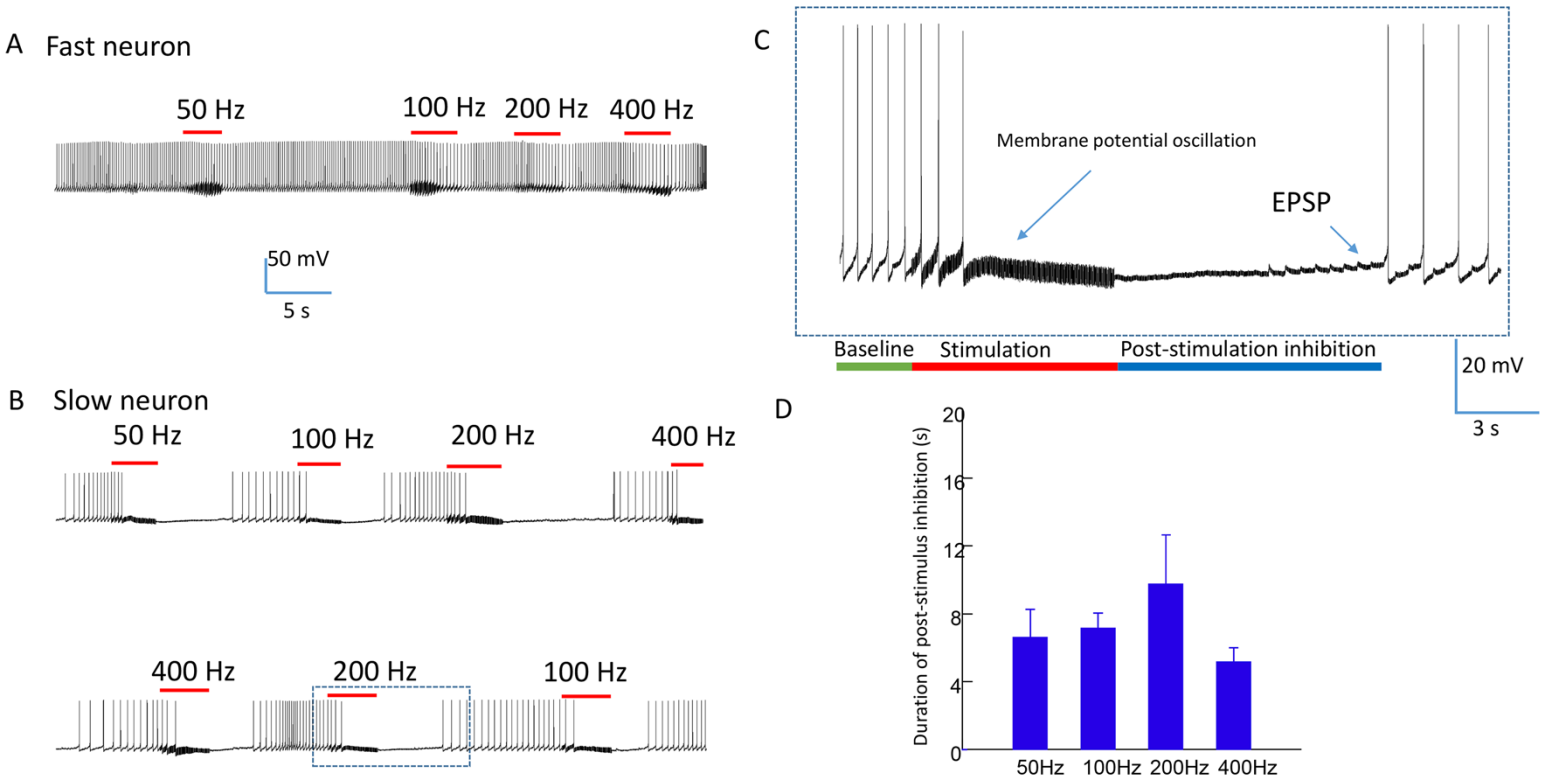
Carry-over (post-stimulation inhibition) effects after high frequency magnetic stimulation in spontaneously firing neurons. (**A**) Neural activity was not blocked by high frequency magnetic stimulation (50–400 Hz) in neurons firing at high rate (> 3 Hz). (**B**) Neural activity was completely blocked by high frequency magnetic stimulation (50 Hz-400 Hz) in neurons firing at a relatively low rate (< 2 Hz). (**C**) Expanded trace from (**B**) (dashed rectangular box) demonstrates the oscillation of membrane potential during high frequency magnetic stimulation, and post-stimulation inhibition. The neuron recovered from the carry-over effects by receiving excitatory synaptic input. EPSP: excitatory postsynaptic potential. (**D**) Duration of carry-over, post-stimulus inhibition was independent of the tested frequency (50–400 Hz).

Here, we further tested the effects of a range of stimulus frequencies (50–400 Hz) in neural inhibition. Figure 3 demonstrates a neuron that fires spontaneously between 1 and 3 Hz. When a steady rate of firing was observed, we applied 6–8 s of magnetic stimulation to the ganglion neuron. Consistent with previous work^12^, when the neuron fired action potentials at a high rate (> 3 Hz), the neuron was not inhibited by the magnetic field under the various tested frequencies (Fig. 3A). Instead, when the neuron fired action potentials at a lower rate (< 2 Hz), the neuron was instantly and reversibly blocked by the magnetic field at various frequencies between 50 and 400 Hz (Fig. 3B).

Interestingly, after the coil stimulation was terminated, we observed a carry-over, post-stimulation inhibition effect. The neurons could resume firing after 5–10 s of carry-over inhibition (Fig. 3B). A close examination of the membrane potential revealed that the high frequency magnetic stimulation generated an oscillation of the membrane potential in the cell, which led to the inhibition of neuronal activity. During carry-over inhibition, the neuron resumed firing after receiving excitatory synaptic input (Fig. 3C).

To explore whether carry-over effects could be dependent on the stimulus frequency, we measured the duration of the carry-over period under a spectrum of tested frequencies (50 Hz, 100 Hz, 200 Hz, and 400 Hz) in five neurons. Each experimental frequency resulted in post-stimulus inhibition (Fig. 3D). One-way repeated measures ANOVA revealed that a field frequency between 50 and 400 Hz was not a strong factor in determining the duration of post-inhibitory effects (*F*_(3, 14)_ = 1.37, p = 0.29). Overall, magnetic stimulation produced a 7.03 ± 0.83 s duration of post-stimulation inhibition before the neuron could resume firing.

### Magnetic stimulation generated carry-over, post-stimulation inhibition in neurons driven by depolarization current

**Figure 4:**
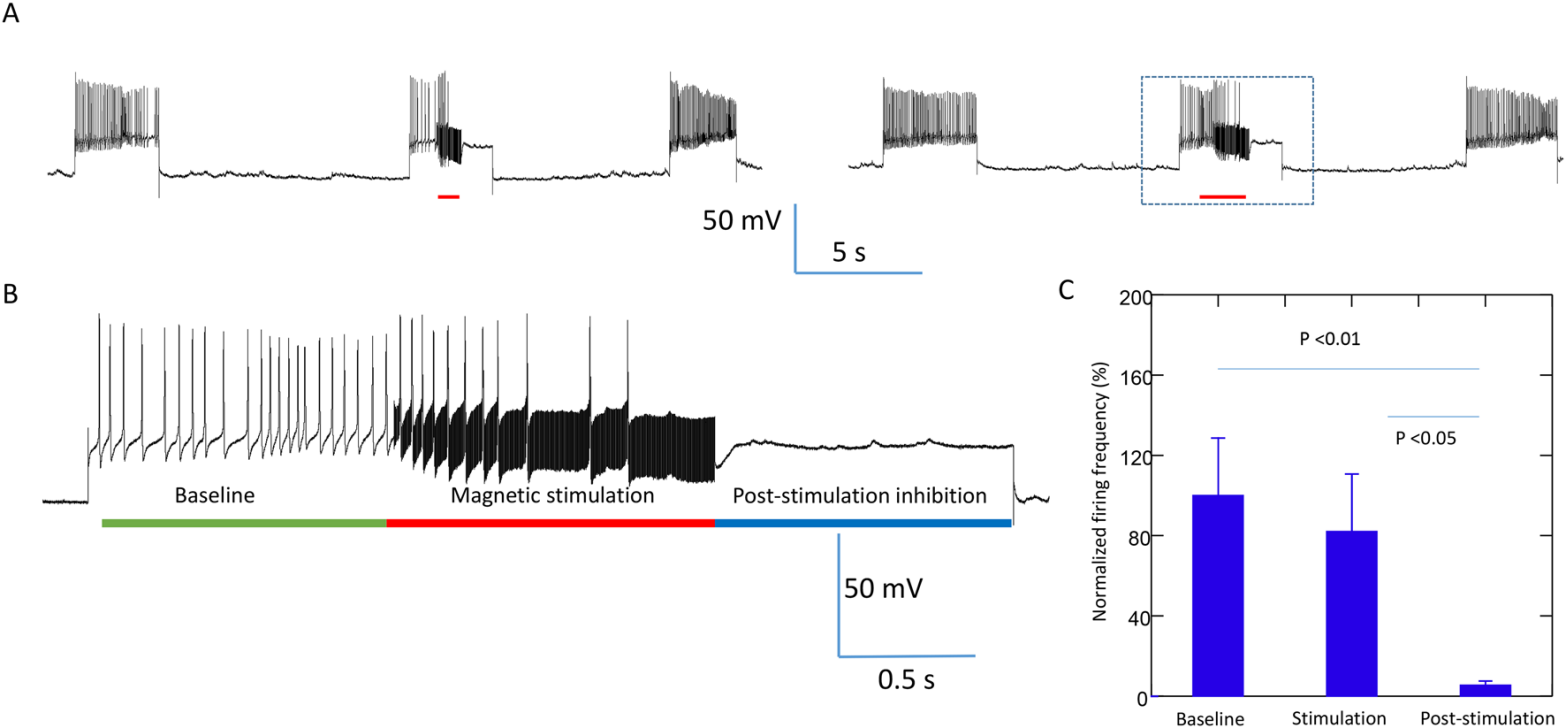
Carry-over (post-stimulation inhibition) effects after high frequency magnetic stimulation in neurons with evoked activity. Neuronal activity was elicited with intracellularly injected depolarization currents, which was blocked by 400 Hz magnetic stimulation. (**A**) Represented traces demonstrate that continuous neural firing during membrane depolarization was blocked by the magnetic field. (**B**) An expanded trace demonstrates the firing of the neuron (baseline), oscillation of the membrane potential during magnetic stimulation followed by the ultimate blockage of the neural activity, and the complete quiescent of the neuron after the magnetic field was terminated, although the depolarization current was still injected into the cell. (**C**) Statistics from multiple trials demonstrate the frequency of neural firing before, during, and after magnetic stimulation. Data was normalized to the baseline firing frequency.

Aside from neurons that can generate spontaneous activity, neurons that are quiescent in the buccal ganglion could be evoked to fire action potentials with the injection of positive currents. In the first protocol, constant electric currents were injected into the quiescent buccal neurons for approximately 5 s. When neurons reached a stable firing frequency, a 400 Hz magnetic field was applied to the neuron. Consistent with our previous study^4^, we observed these neurons to be completely inhibited within 1 s after the onset of the magnetic stimulation (Fig. 4A), even if the depolarization current was continuously applied to the soma (n = 9). Interestingly, when the coil stimulation was withdrawn, the neurons could not recover from complete inhibition, even if the cell membrane was significantly depolarized by the injected current. We measured the firing frequency of the neuron before, during, and after the magnetic stimulation. The firing frequency of the neuron started to decrease during magnetic stimulation, until a complete blockage was achieved. Paired t-test indicated that, post-stimulation, the firing frequency of the neuron was significantly (p = 0.009) lower than that before the magnetic stimulation (only 5.39% ± 2.15% of that of the pre-stimulation frequency), even when the same amount of current was injected into the soma for membrane depolarization.

**Figure 5:**
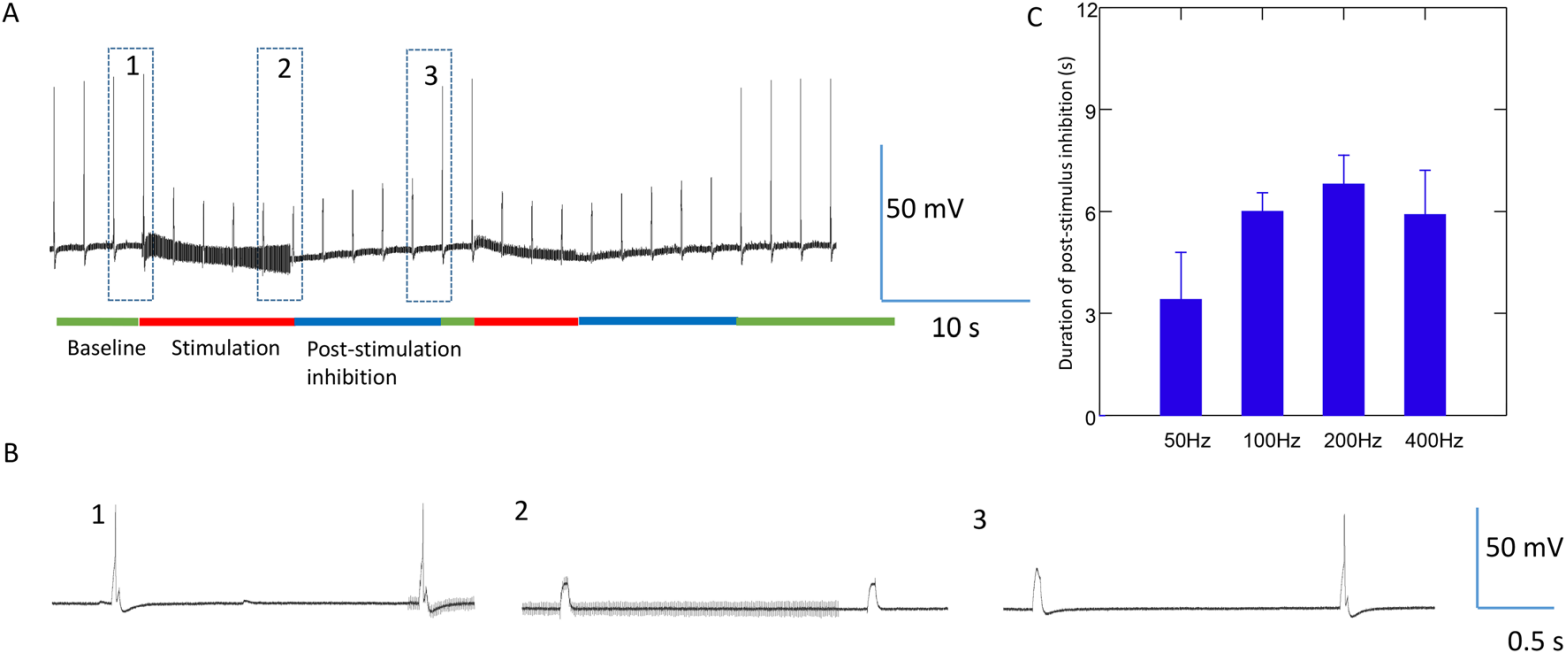
Carry-over effects observed when the neuron was evoked to fire at a constant frequency. The neuron was elicited to fire a single action potential by short current pulses at 0.5 Hz. The coil stimulated the soma at 400 Hz for approximately 7–10 s (red bars). (**A**) Magnetic stimulation inhibited the soma firing, followed by a carry-over, post-stimulation inhibition. (**B**) Expanded traces indicted by the numbered (1, 2, and 3) rectangles in (**A**). (**C**) Duration of carry-over, post stimulation inhibition induced by magnetic field of various field frequencies.

In the second protocol, we delivered short current pulses to the neuron at a fixed frequency (0.5 Hz, Fig. 5). Duration of these pulses was adjusted so that each depolarization pulse could trigger one single action potential from the recorded neuron. When high frequency magnetic stimulation (400 Hz) was applied to the soma, consistent with our previous study^4^, the coil stimulation (10 s) rapidly and reversibly blocked the 0.5 Hz firing in the recorded neurons (n = 7). Inhibition of the neuron persisted after the withdrawal of the magnetic field, even if the electric pulses were regularly injected into the soma. We observed a carry-over effect of 5.9 ± 1.3 s, before the neuron could resume firing again.

To test the effects of magnetic field frequency on the duration of carry-over post-inhibition, we applied a spectrum of stimulation frequencies (50 Hz, 100 Hz, 200 Hz, and 400 Hz) to the neuron. We did not notice any significant impact of these different stimulation protocols on the carry-over duration (F(3,24) = 0.815, p = 0.498, Fig. 5C).

### Trans-sheath magnetic stimulation generated carry-over, post-stimulation inhibition

One major advantage of magnetic stimulation is that the magnetic field can penetrate the dielectric biological material without attenuation, allowing control of a neuron noninvasively^13^. Therefore, the miniature coil should be equally effective in stimulating the neurons in the intact buccal ganglion, under the cover of the ganglion sheath. We hypothesized that magnetic stimulation across the ganglion sheath could induce carry-over effects. Unfortunately, due to thickness of the ganglion sheath, it was not feasible to monitor neuronal activity using the intracellular technique. To solve this problem, we used the extracellular single cell recording technology.

**Figure 6:**
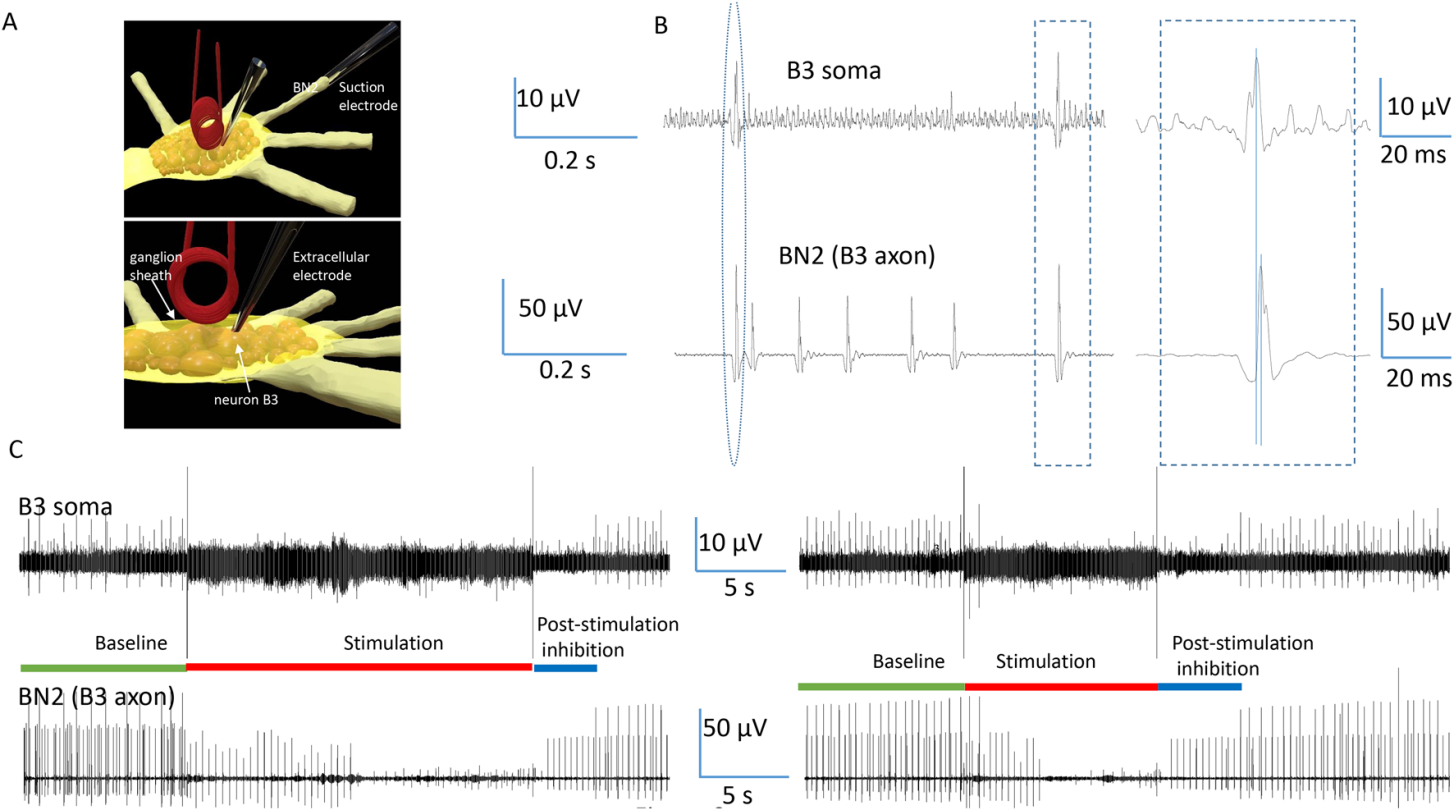
Carry-over effects observed after trans-sheath magnetic stimulation. (**A**) Activity of the jaw motor neuron B3 was recorded from the soma and from the axon, respectively. One extracellular electrode was positioned on the ganglion sheath, on top of the B3 soma. One suction electrode was applied to the buccal nerve II (BN2), to record the axonal activity from the B3 neuron. (**B**) One-to-one relationship between the B3 soma activity and the largest BN2 activity (B3 axonal activity). (**C**) 400 Hz magnetic stimulation inhibited the B3 activities and carry-over, post-stimulation inhibition was observed in this neuron.

The buccal ganglion contains the cell bodies of several identified motor neurons (B3, B6, B9, and B10), whose axons travel through buccal nerve II (BN2)^37^ and innervate the I1/I3 jaw muscle to generate the feeding behavior of *Aplysia*^35,36^. Among these neurons, B3 motor neurons have the largest size^35,36^. The firing of these neurons generates large spikes in the extracellular recordings from BN2^35,38^. We, therefore, applied an extracellular electrode on top of the ganglion sheath, right above the B3 soma, to record B3 activity. We also applied another suction electrode on the distal end of BN2 (Fig. 6A^39^), which was approximately 2 cm away from the coil and the ganglion, to monitor the B3 axonal action potential. Since the coil-induced electric fields decay quickly with distance^40,41^, and a tight suction could eliminate a significant amount of magnetic interference^39^, this technology recorded minimal noise during coil stimulation. More importantly, it monitored the output of the motor neurons whose cell bodies were under magnetic stimulation.

Figure 6B demonstrates the dual recording traces. There is a one-to-one relationship between the largest spikes in the BN2 recording and the B3 activity (blue, dashed circle/rectangle in Fig. 6B). These somatic activities preceded the large spikes in BN2 by approximately 3 ms, suggesting the action potentials in the B3 neuron were initiated from the soma. The electrode on the buccal ganglion also recorded some small spikes, likely due to the activity of the neurons adjacent to the B3 neuron.

During high frequency magnetic stimulation, the somatic activity in B3 was completely inhibited, as were the large spikes (B3 axonal activity) in BN2. After the termination of the magnetic stimulation, we observed carry-over, post-stimulation inhibition in the B3 neuron (Fig. 6C), which lasted for 4.04 ± 0.32 s (n = 5).

### NEURON simulation confirmed that high frequency magnetic inhibition led to carry-over, post-stimulation inhibition

To simulate the carry-over effects evoked by the high frequency magnetic field, we utilized our published biophysical model that computed the magnetically induced electric field in the vicinity of the neuron^4^. We then combined the preexisting model with a multi-compartment NEURON model that simulates a single *Aplysia* neuron^4,12^. Figure 2 demonstrates the model configuration, including a spherical soma and a cylindrical axon. The magnetically induced electric field contains both spatial and temporal profiles. For spatial distribution, we calculated the magnetically induced electric field around the neuron, based on the coil parameters provided by the vendor and the parameters used in the neural stimulation experiments.

Previously, we have found that when high frequency voltage pulses were delivered into the miniature coil, the magnetically induced electric field was biphasic, with the first phase corresponding to the onset of the coil current, and the second phase corresponding with the offset phase of the coil current^41,42^. This observation was also confirmed with the mathematic derivation of the electric field (Eqs. 3–6). Therefore, for temporal information, we modeled the electric field as 400 Hz biphasic pulses, which was then applied to the cell model.

Previous studies have demonstrated that electric or magnetic stimulation could affect ion channel functions. Among these channels, voltage-dependent sodium channels and potassium channels are the most studied since they directly contribute to the initiation and sustainability of the action potential. For example, high frequency stimulation using monophasic electric current was shown to depolarize the membrane, inactivate sodium channels, and impair the mechanisms of neuronal firing^43^. Low frequency magnetic simulation altered the kinematics of sodium and potassium channels in hippocampal pyramidal neurons^44^. We, therefore, include these Hodgkin–Huxley (H–H) type channel mechanisms in our simulation.

**Figure 7:**
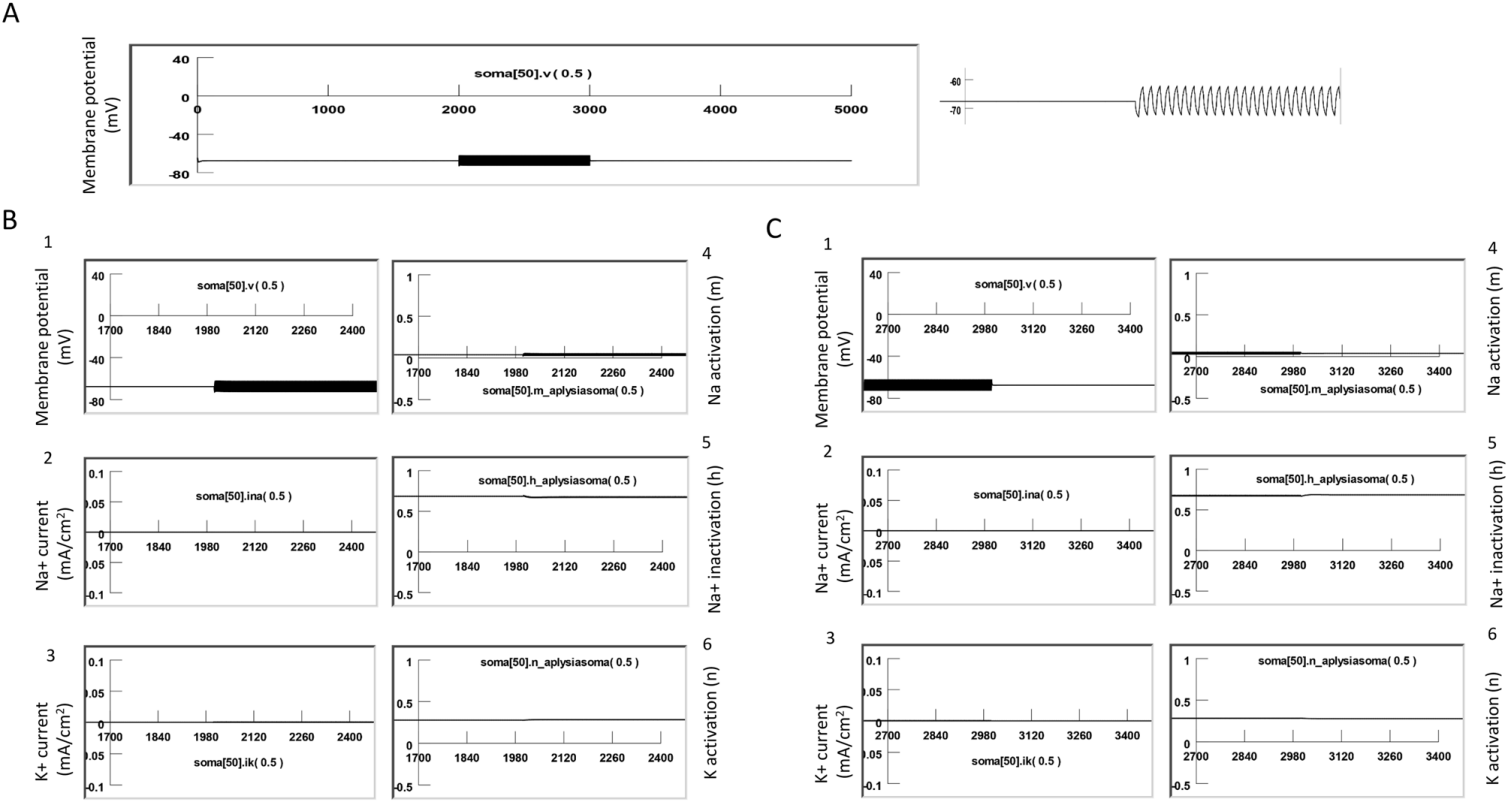
High frequency magnetic field resulted in oscillation of membrane potential and minimal ion channel dynamic changes in a quiescent neuron. (**A**) Oscillation of membrane potential during stimulation. (**B**) Membrane potential (1), Na current (2), K current (3), Na activation (4), Na inactivation (5), and K activation (6) at the start of the high frequency magnetic stimulation. (**C**) Membrane potential (1), Na current (2), K current (3), Na activation (4), Na inactivation (5), and K activation (6) at the end of the high frequency magnetic stimulation.

Figure 7 demonstrates the cellular response to the magnetic stimulation when the neuron was quiescent. In the absence of coil stimulation, the membrane was at resting potential (− 65 mV). We plotted the membrane potential, Na+ current (INa), K+ current (IK), Na channel activation (m), Na channel inactivation (h), and K channel activation (n) when the high frequency magnetic field was applied to the neuron. In agreeance with our electrophysiology observations (Figs. 3, 4, 5), we observed oscillation of the membrane potential in response to the high frequency magnetic stimulation. The magnetic stimulation was not associated with significant changes in the channel current or channel dynamics of the quiescent neuron. However, since m, h, and n are parameters that depend on the membrane voltage^45^, high frequency oscillation was also observed in these state variables during the magnetic stimulation.

**Figure 8:**
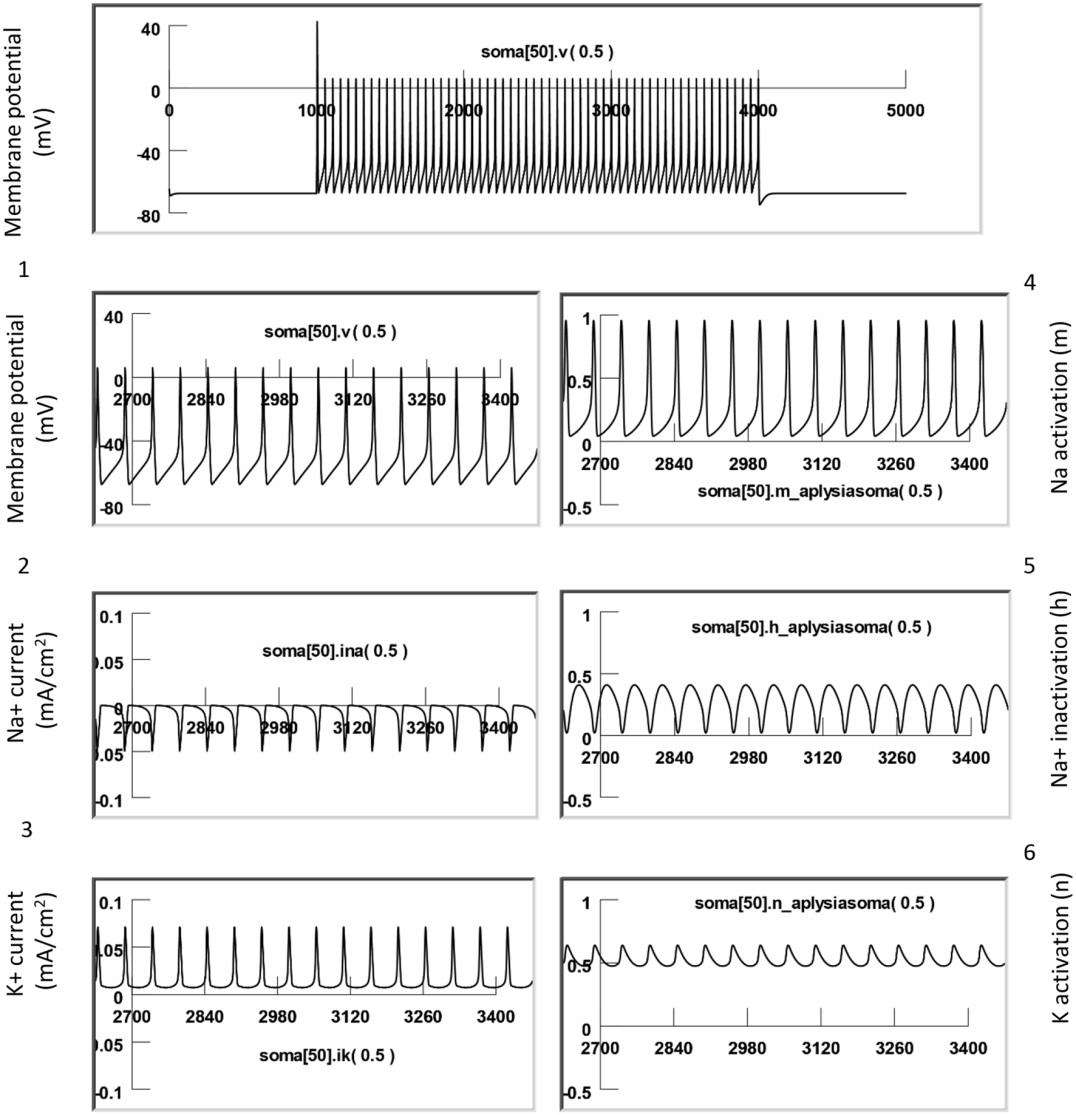
Neural activity and underlying channel dynamics. The modeled neuron was driven by an 18 nA positive current to fire at around 10 Hz at 20 °C. This figure illustrates the membrane potential (1), Na current (2), K current (3), Na activation (4), Na inactivation (5), and K activation (6) during a train of action potentials.

To simulate magnetic stimulation on the activated neurons, we injected depolarization currents to the modeled neuron to elicit action potentials. Figure 8 demonstrates the dynamic changes of channel activities underlying the action potentials. Here, the sodium channel was sufficiently de-inactivated (h = 0.4) before the firing of each action potential. This allowed a sufficient activation of the sodium channels (m = 0.95) to produce a large inward sodium current (INa) and depolarization of the membrane for spiking. Meanwhile, activation of the potassium channels was substantial (n = 0.65), and a large inward potassium current was observed during the falling phase of the action potentials.

**Figure 9:**
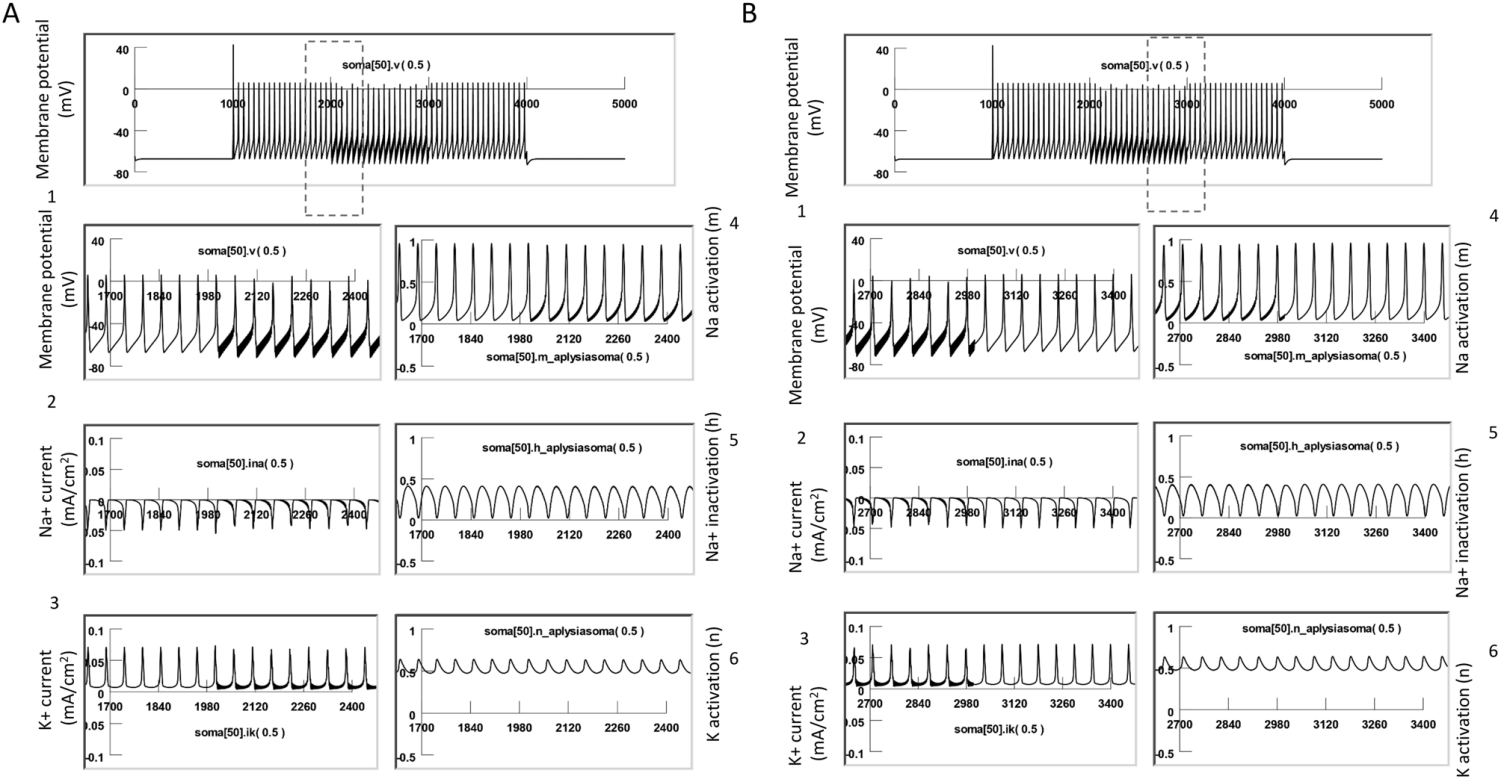
Subthreshold stimulation with magnetic field. The modeled neuron was fired by an intracellularly injected current (18 nA) to generate a train of action potentials, and the magnetic stimulation was applied with a subthreshold intensity (84.5% of the threshold). The model was run at 20 °C. This figure illustrates the membrane potential (1), Na current (2), K current (3), Na activation (4), Na inactivation (5), and K activation (6). (**A**) Start of magnetic stimulation. (**B**) Post-stimulation. Magnetic stimulation has minimal effects on membrane excitation and the underlying state variables that control channel dynamics.

We then applied 400 Hz stimulation pulses to the coil, as in the electrophysiology experiments. Figure 9 demonstrates the response of a neuron under subthreshold magnetic stimulation. The model neuron was fired at around 10 Hz by an intracellularly injected step current (18 nA), as seen in the electrophysiology experiments (Fig. 4B). When the high frequency magnetic stimulation was applied to the neuron at a subthreshold intensity (82.5% of the threshold intensity), a membrane oscillation was introduced during the firing of the action potentials. We also observed oscillation in m, h, and n parameters. These state variables could reach their expected values to sustain the action potentials, as depicted in Fig. 8.

**Figure 10:**
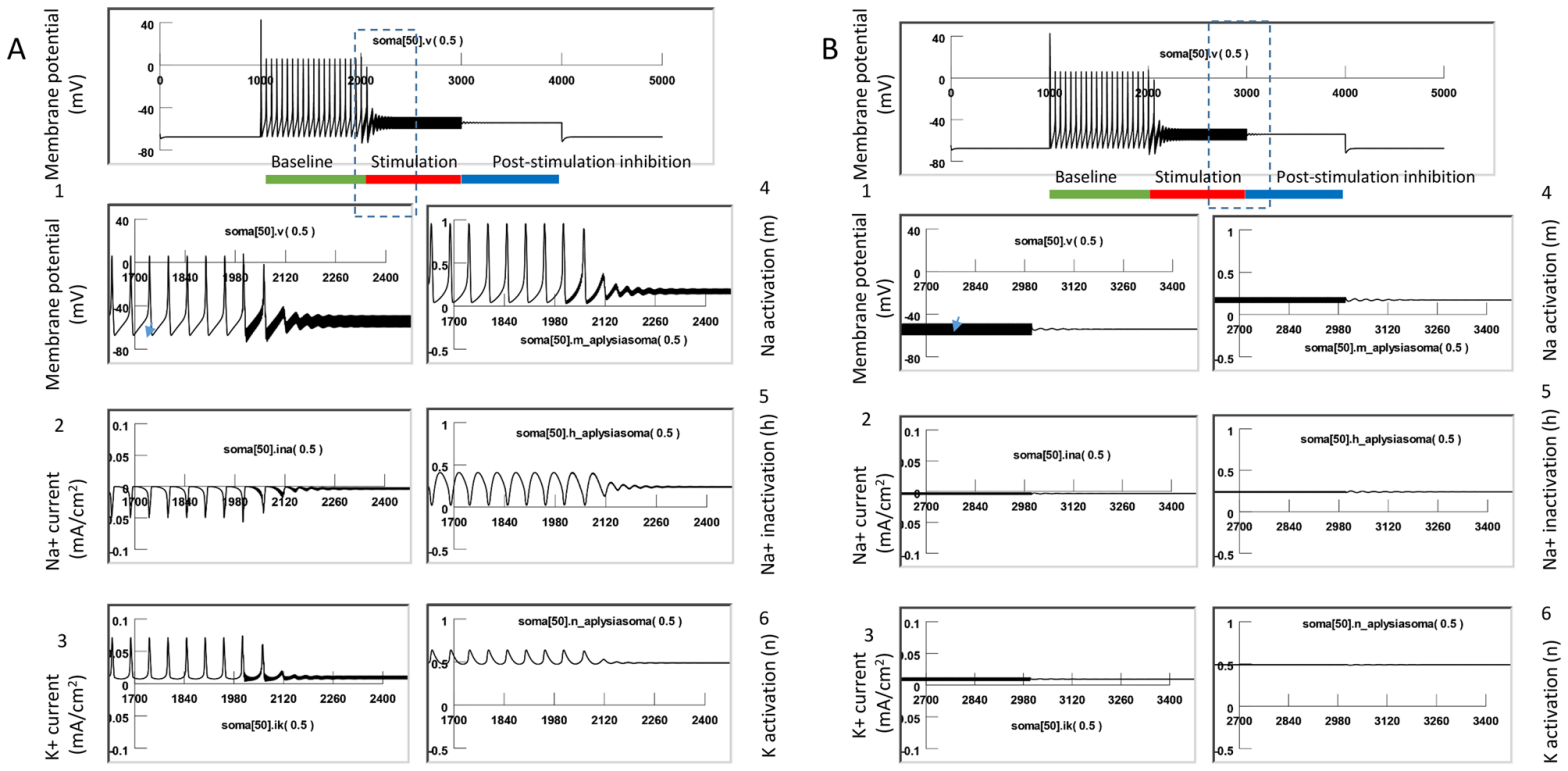
Supra-threshold stimulation with magnetic field. The model neuron was fired by an intracellularly injected current (18 nA) to generate a train of action potentials, and the magnetic stimulation was applied with a suprathreshold intensity (101.5% of threshold). This figure illustrates the membrane potential (1), Na current (2), K current (3), Na activation (4), Na inactivation (5), and K activation (6). (**A**) Start of magnetic stimulation. Magnetic field blocked the action potential by preventing the full activation (m) and full inactivation (h) of the Na channel, and the full activation of the K channel (n). (**B**) Post-stimulation. The fast oscillation in membrane potentials, as well as the slight oscillation in the m, h, and n parameters, stopped. There was a slight change in the m and h values right after magnetic stimulation, but these changes were insufficient to trigger action potentials, thereby surrendering the neuron to the carry-over period for inhibition.

Figure 10 demonstrates suprathreshold stimulation with the magnetic field. 400 Hz stimulation resulted in neural inhibition and carry-over, post-stimulation inhibition. We examined the dynamics of the ion channels. At the start of magnetic stimulation (Fig. 10A), the fast influx of the sodium current was interrupted and diminished. The magnetic stimulation prevented the activation of the sodium channels; state variable m was observed to decrease from 0.95 to 0.2. In addition, the magnetic stimulation prevented sufficient de-inactivation of the sodium channels; state variable h decreased from 0.4 to 0.25. Since the conductivity of the sodium channel is defined by m^3^h^45^, this result suggests that sodium channel conductance was reduced under magnetic stimulation, preventing the ignition of an action potential in the neuron. Meanwhile, the potassium channels (n = 0.5) were unable to be activated, leading to a diminished outward potassium current. A constant potassium current was observed due to continued depolarization of the membrane.

Immediately after the termination of the magnetic field (Fig. 10B), the fast oscillation in the membrane potential, as well as the slight oscillation in the m, h, and n parameters, stopped. There was some slow oscillation in the m and h traces right after magnetic stimulation, but they were not sufficient to trigger action potentials. Instead, when the high frequency magnetic field was withdrawn, the m, h, and n parameters quickly reached a steady state. The sodium channel conductivity was maintained to be low, and the neuron entered the carry-over period for inhibition.

In summary, the carry-over effects observed after high frequency magnetic stimulation were due to the field-induced, fast oscillation of the membrane potential, which impaired the normal ion channel dynamics that were responsible for the initiation and sustaining of the action potential.

### Voltage-clamp simulation revealed reduced sodium channel conductance and enhanced potassium channel conductance during the carry-over period

To further investigate the impact of magnetic stimulation on channel properties during carry-over post-inhibition, we performed a voltage clamp experiment using the NEURON model.

**Figure 11:**
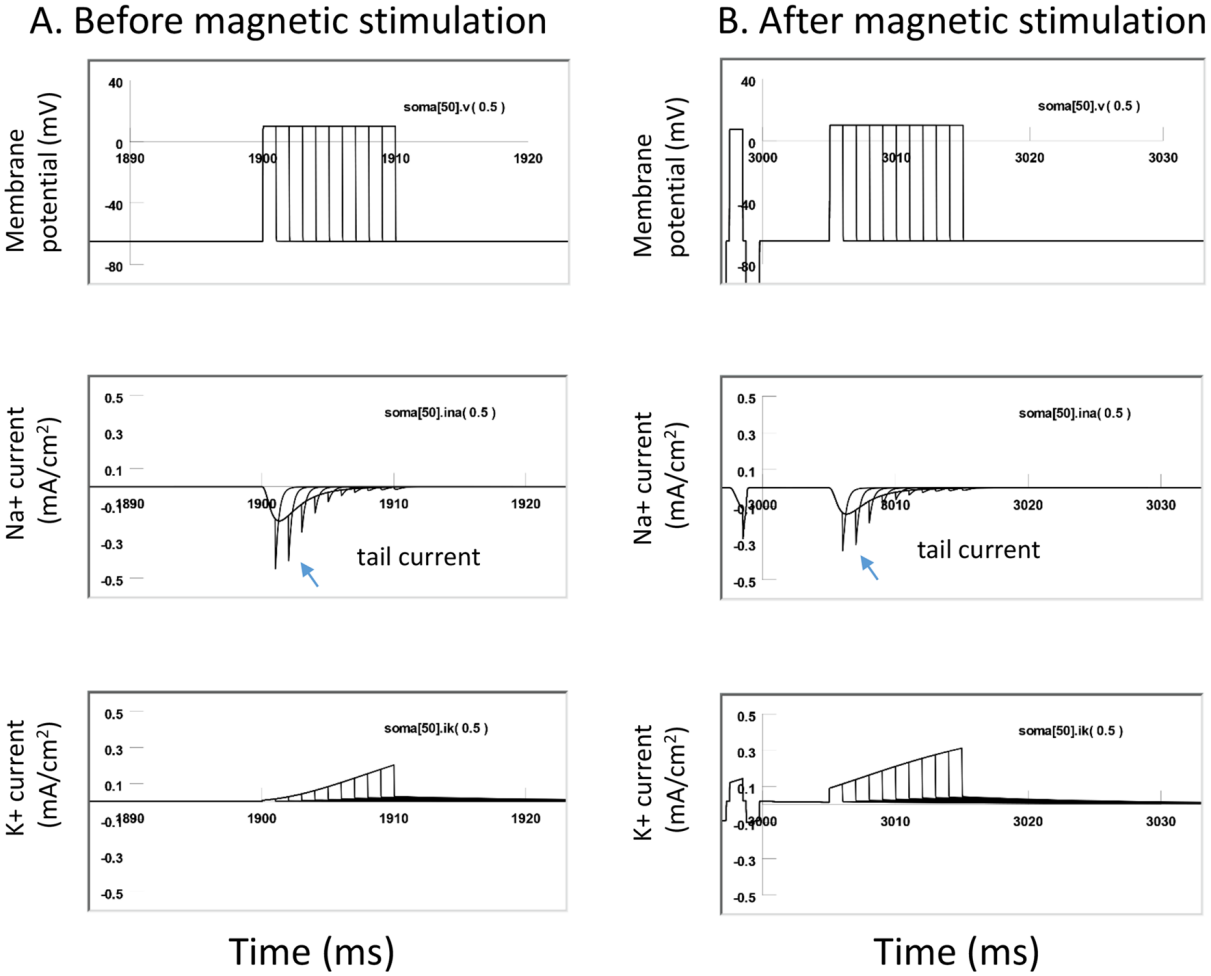
Carry-over effects were mediated by the altered the sodium and potassium channel conductance after magnetic stimulation. The sodium and potassium currents were measured and compared before and after the magnetic stimulation in a voltage clamp experiment (NEURON simulation). (**A**) Before magnetic stimulation. The membrane potential was clamped from − 65 to 10 mV with varying duration (from 1 to 10 ms), producing the inward sodium current and outward potassium current. Note the sodium tail currents produced at the end of the voltage steps. (**B**) The same voltage clamp protocol was applied to the neuron immediately after the cell had been stimulated with 400 Hz magnetic stimulation for 3000 ms. In comparison with (**A**), the INa tail current was significantly decreased, and IK significantly increased after magnetic stimulation.

Figure 11A demonstrates the measurement of INa and IK before and after the high frequency magnetic stimulation (i.e., during the carry-over period). The membrane potential of the middle segment of the soma (Soma [50]) was clamped from − 65 to 10 mV for 10 ms (Fig. 11A), which led to a fast inward sodium current (INa), followed by a delayed outward potassium current (IK). The maximal INa was 0.19 mA/cm^2^, and the maximal IK was 0.20 mA/cm^2^, respectively. By varying the duration of holding potentials (1–10 ms, with 1 ms increments), we recorded the sodium tail currents. Generated by the sudden termination of the test pulse and the instant increase of the electrical driving force on the sodium ions, the time profile of the tail current provided an excellent indication of sodium channel conductance (gNa) during the clamp pulse^45^.

We then applied the magnetic stimulation (400 Hz) to the cell for 3000 ms. Immediately after the termination of magnetic stimulation, we applied the same voltage clamp protocol to the neuron (Fig. 11B). The INa was significantly decreased after magnetic stimulation, and IK significantly increased. The maximal INa was reduced to 0.13 mA/cm^2^, yielding a 31.6% decrease from the pre-stimulation value under a 10 ms voltage clamp. The maximal IK was increased to 0.31 mA/cm^2^, yielding a 55% increase from the pre-stimulation value. In addition, the amplitude of the sodium tail currents was significantly decreased by the magnetic stimulation, indicating a reduced gNa during the carry-over, post-stimulation inhibition.

In summary, our voltage-clamp experiment confirmed that, after high frequency magnetic stimulation, ion channel dynamics that support neural excitability become impaired, feathered by the reduced sodium conductance and increased potassium conductance. These changes in channel properties were initiated by the oscillation of membrane potential in the high frequency magnetic field. Impairment of ion channel dynamics instigated a deficit in the action potential generation mechanism and, eventually, led to carry-over inhibition.

### Controlling carry-over effects after high frequency magnetic stimulation

Results thus far suggest that neurons acquiesce to the carry-over period due to impaired ion channel dynamics that support the initiation of action potentials after high frequency magnetic stimulation. Since these ion channel dynamics are dependent on the membrane potential, perturbation of the membrane potential while neurons are in the carry-over, inhibitory state, could provide a method to control the carry-over, post-stimulation inhibition.

#### Carry-over effects could be controlled by providing neurons with extra excitatory drive

NEURON simulation suggested that the observed decrease in gNa was due to insufficient activation (m) of the sodium channels (Fig. 10). On the other hand, recording from spontaneously firing neurons suggested that carry-over effects are minimized when the neurons are driven by additional excitatory synaptic input (Fig. 3C). Therefore, we hypothesized that the carry-over inhibition could be shortened by membrane depolarization, which would force a greater sodium current to elicit action potentials.

**Figure 12:**
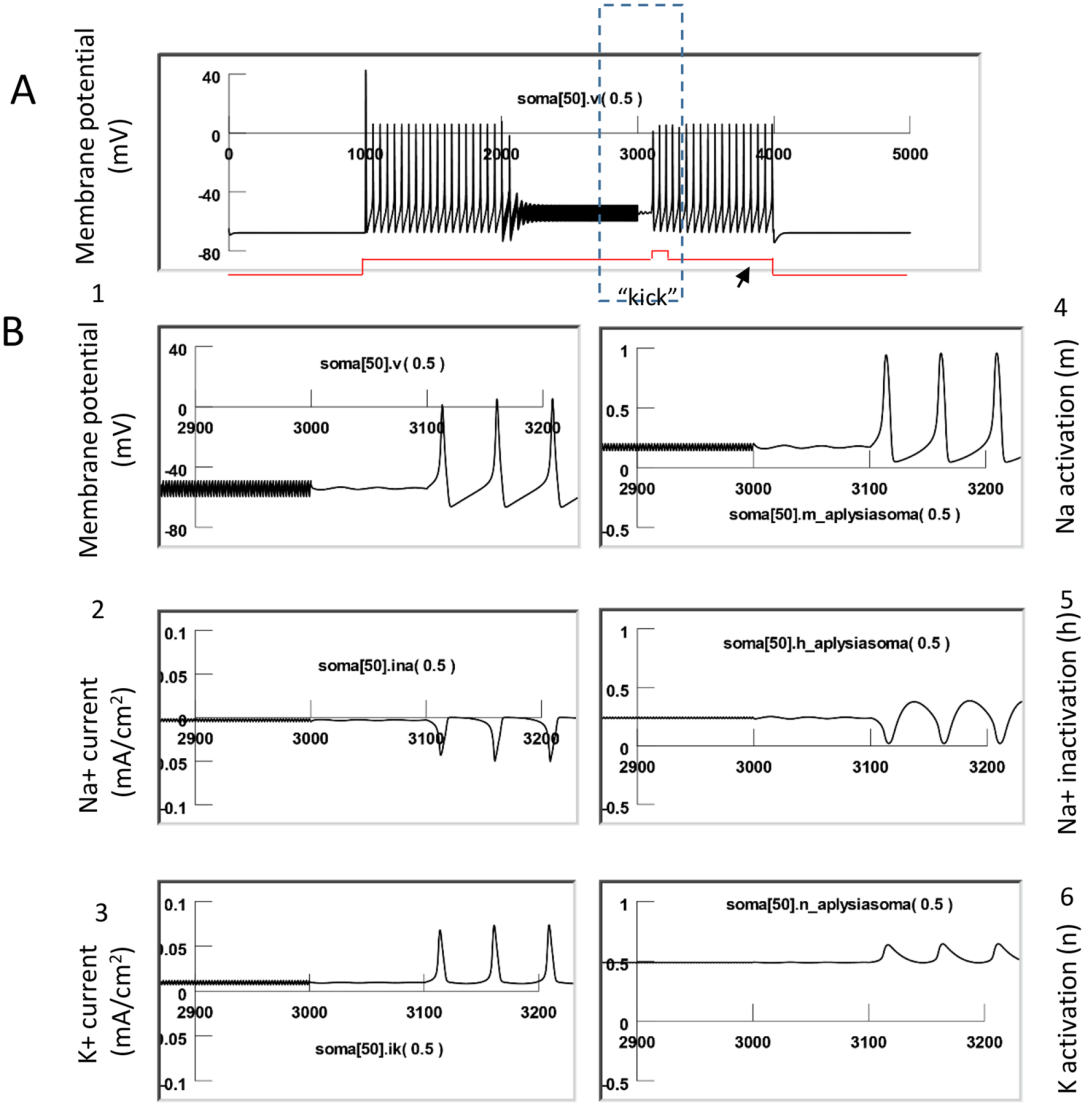
Carry-over effects eliminated by temporal enhancement of excitatory drive to the neuron. (**A**) The neuron was driven to fire a sequence of action potentials with a long depolarization step (18 nA). High frequency magnetic stimulation blocked the action potentials and carry-over effects were observed (also see Fig. 10B). A short “kick” current (0.1 s in duration) was applied to the neuron (with intensity of 2 nA, in addition to the 18 nA depolarization current) to eliminate the carry-over period, and the neuron could fire again under the same depolarization current. (**B**) Membrane voltage (1), INa (2), IK (3), and m (4), h (5), and n (6) parameters correlated with the “kick” current.

In Fig. 12, the modeled neuron was triggered to fire action potentials with the injection of a constant depolarization current. A one second, 400 Hz magnetic stimulation was then applied to the neuron to inhibit it, and the carry-over, post-stimulation inhibition was observed. We then applied a “kick” pulse (200 ms in duration) into the neuron, which provided a short stimulus with slightly higher (2 nA) intensity. The neuron started to fire, and this excitation maintained even after withdrawing the “kick” pulse. A close look at the ion channel dynamics suggested that the “kick” current provided the necessary boost to activate the sodium channels, reflected by the significant increase in the state variable, m. Once the neuron resumed firing, the H–H mechanisms ensured the sustained firing of the neuron after the termination of the short “kick” current.

**Figure 13:**
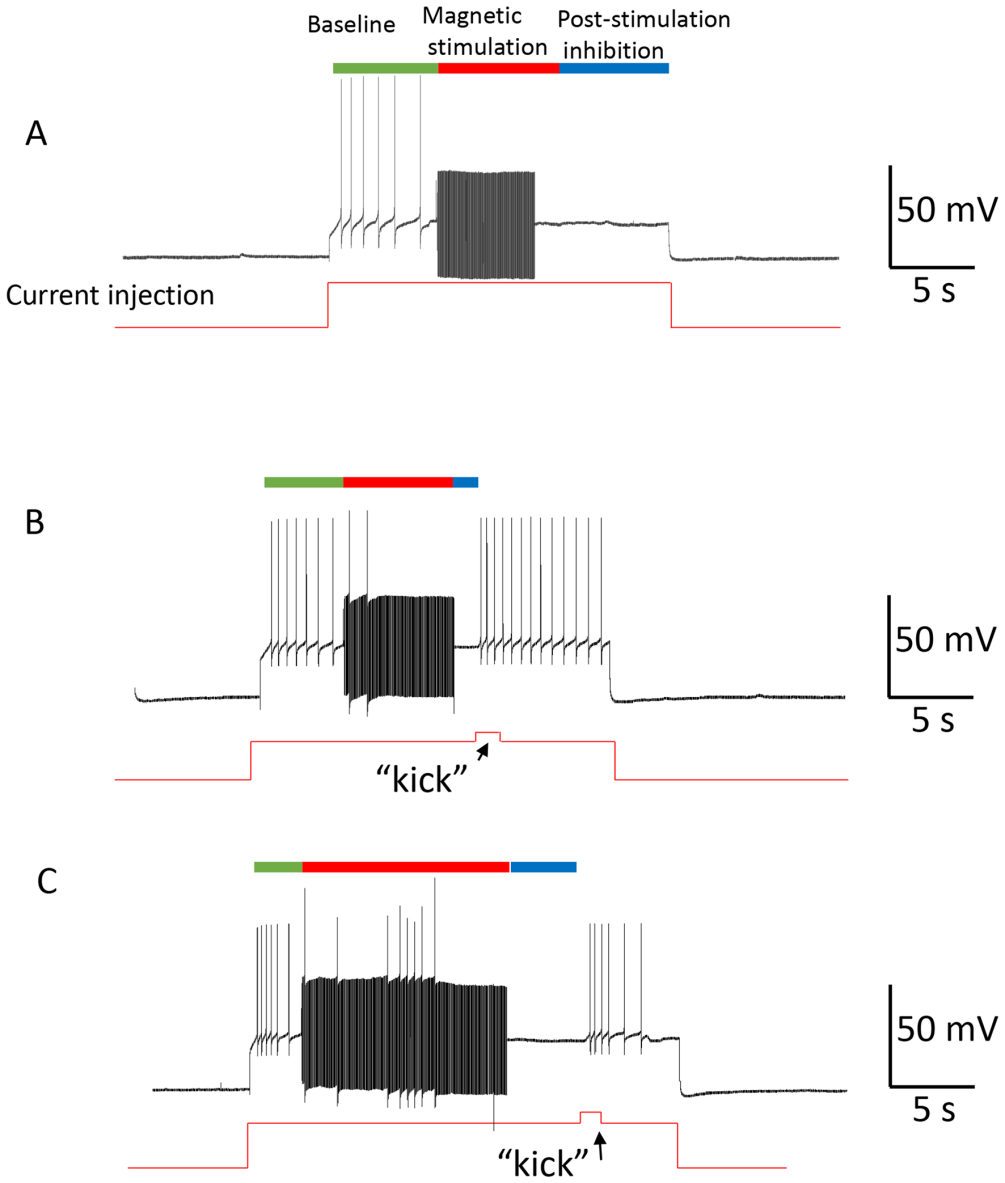
Electrophysiological confirmation of the elimination of carry-over, post-stimulation inhibition with a short “kick” stimulus. (**A**) Carry-over effects observed after high frequency magnetic stimulation. A depolarization current (5 nA) was used to depolarize the membrane to elicit a train of action potentials (Vm = 66 mV), which was inhibited by 400 Hz magnetic stimulation for 6 s, followed by a post-stimulus inhibition period. (**B**) When a short “kick” current (1 nA for approximately 1 s) was injected into the cell membrane, the neuron was able to resume firing action potentials. (**C**) A relatively late “kick” current was equally efficient in bringing the neuron out of the carry-over inhibitory state.

To directly test the efficacy of the “kick” current in eliminating the carry-over, post-stimulation inhibition, we applied an intracellular recording to the buccal neurons. We elicited a train of action potentials using a step current (Fig. 13). A 400 Hz magnetic stimulation eliminated the action potentials and the neuron fell into carry-over post-inhibition (Fig. 13A). We then applied a short “kick” current to the neuron, which elicited the neuron to fire again (Fig. 13B, n = 4). The exact moment at which the neuron resumed its firing capability was dependent on the timing of the “kick” current (Fig. 13C).

#### Carry-over effects could be controlled by temporal removal of the excessive excitatory drive to the neuron

NEURON simulation also suggested that the observed decrease in gNa was due to insufficient de-inactivation (h) of the sodium channels (Fig. 10). Therefore, we hypothesized that it is possible to increase the chance for neural firing during the carry-over inhibition by eliminating the excessive excitatory drive to the cell. This would increase the availability of the sodium channels for the resumed initiation of action potentials.

**Figure 14:**
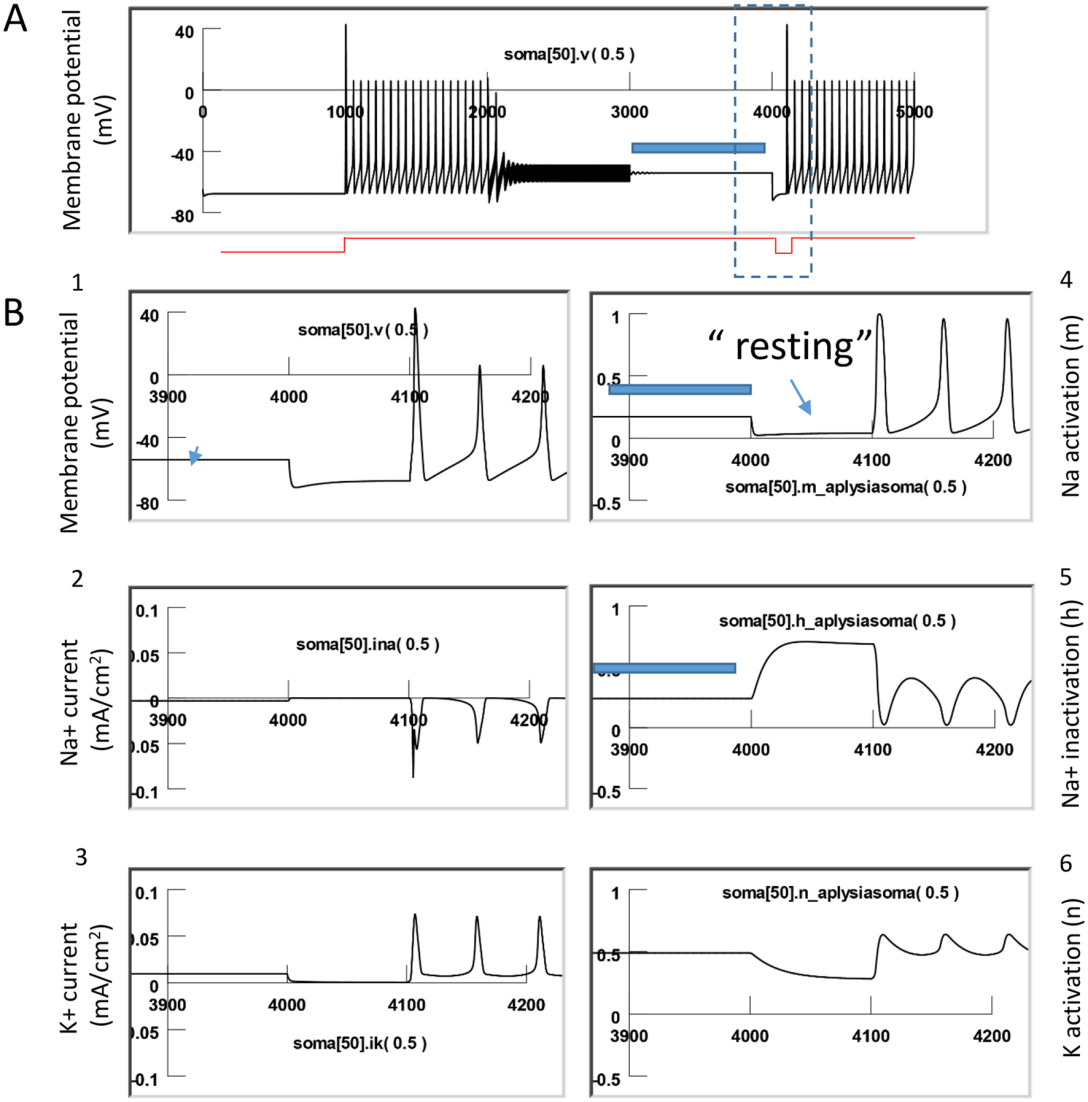
Carry-over effects eliminated by temporal removal of excessive excitatory drive to the neuron. (**A**) The neuron was driven to fire a sequence of action potentials with a long depolarization step (18 nA). High frequency magnetic stimulation blocked these action potentials, followed by a carry-over period. A brief withdrawal (0.1 s) of the depolarization current eliminated the carry-over period, and the neuron was observed to fire again under the same depolarization current. (**B**) Membrane voltage (1), INa (2), IK (3) and m (4), h (5), and n (6) parameters during the termination of the excessive depolarizing drive to the neuron.

In Fig. 14, the modeled neuron was triggered to fire action potentials with the injection of a constant depolarization current. A one second, 400 Hz magnetic stimulation was then applied to the neuron to inhibit it. The magnetic stimulation also induced the neuron into the post-stimulation inhibition state. We then withdrew the depolarization current for a very short period (100 ms). This 100 ms “resting” period allowed the de-inactivation of the sodium channels and the h value significantly increased, indicating that more sodium channels became available for activation. Although the m value decreased slightly during the “resting” period, the overall gNa (equal to m^3^h^45^) significantly increased, resulting in a large inward INa and initiation of the action potentials.

**Figure 15:**
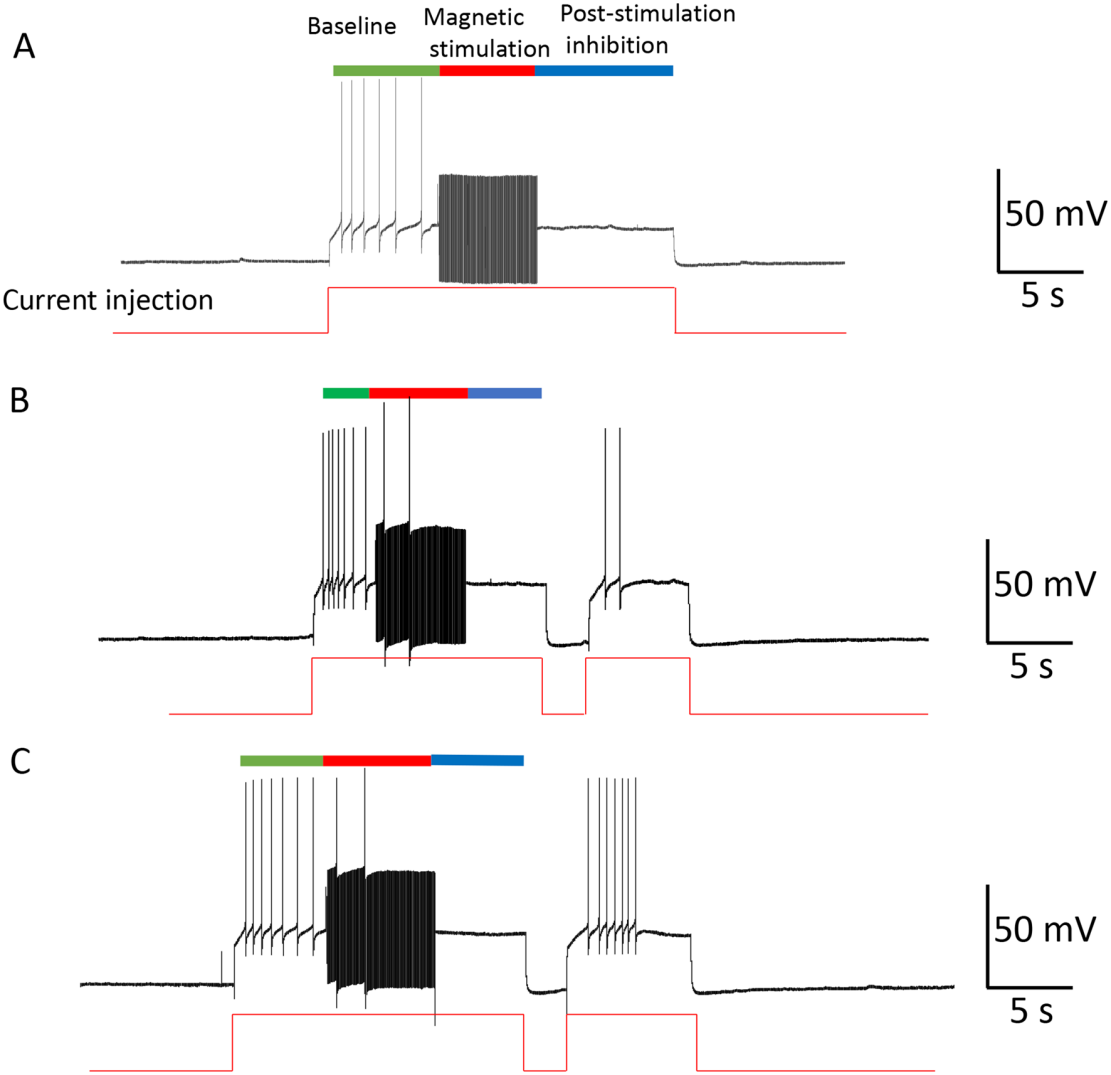
Electrophysiological confirmation of the elimination of carry-over, post-stimulation inhibition with a short “resting.” (**A**) Carry-over effects observed after high frequency magnetic stimulation. A depolarization current (5 nA) was used to depolarize the membrane to elicit a train of action potentials (Vm = 66 mV). 400 Hz magnetic pulses were used to inhibit the neuron for approximately 6 s, and the neuron was observed to fall into a post-stimulus inhibition period. (**B**) Withdrawal of the constant depolarization current allowed the neurons to resume resting membrane potential for a short period, enabling the neuron to escape the carry-over period and resume firing action potentials. (**C**) A relatively late “resting” was equally efficient in bringing the neuron out of the carry-over inhibitory state.

To directly test the idea of temporarily “resting” the membrane to control carry-over inhibition, we applied an intracellular recording from a neuron and used a step current to elicit a train of action potentials (Fig. 15). 400 Hz magnetic stimulation eliminated the action potentials, which was followed by the carry-over, post-stimulation inhibition (Fig. 15A). We then withdrew the depolarization current for a short period (approximately 2 s), and then resumed the injection of the depolarization current. This manipulation rescued the neuron from the carry-over inhibition, and the neuron started to fire continuously (Fig. 15B, n = 6). The exact moment that the neuron resumed its firing capability was dependent on the timing of the temporal “resting,” and the ultimate removal of the excitatory input (Fig. 15C).

## Discussion

In this paper, we investigated carry-over effects after magnetic stimulation at the cellular level, using intracellular electrophysiology and computational modeling. Evidence indicated that carry-over effects are a consequence of the interplay between neuronal properties and the waveform of the stimulus. There are several important discoveries from the work.

First, we reported that high frequency magnetic stimulation led to the carry-over, post-stimulation inhibition of the target neurons. The action potentials in those neurons could be spontaneous (Fig. 3). They also could be induced by a long step depolarization current (Fig. 4) or short pulses of fixed frequency (Fig. 5), which represent different levels and intensities of synaptic drive to the neurons. Coil stimulation was also effective in trans-sheath stimulation (Fig. 6), similar to the practice of transcranial magnetic stimulation (TMS) of the brain. To our knowledge, this work contributes the first cellular characterization of carry-over effects under magnetic stimulation.

When the three in vitro protocols were compared, we found the most prominent carry-over effects when the neuron was depolarized with DC current (Fig. 4). In contrast, neurons that fired spontaneously (Fig. 2), as well as neurons driven by short pulses (Fig. 5), could escape the carry-over period. This result is consistent with those reported when kilohertz electrical stimulation (KES) was used for neural blockage in peripheral nerves^46,47^. Although the mechanism of such effects under KES was not completely clear, it was hypothesized that the DC component delivered by the electrodes could be related to the observed carry-over inhibition^48^. In our study, since the induced electric field was generated via electromagnetic induction, the DC component did not come from the stimulus waveform, but rather from the DC current intracellularly delivered to the neuron. Therefore, one possible solution to control carry-over effects during high frequency electric or magnetic stimulation is by eliminating the DC drive to the membrane.

Second, we explored a range of magnetic field frequencies (50–400 Hz) for single cell stimulation (Fig. 3). Clinically, these frequencies have been used to generate inhibitory effects to neural activity. For example, 60 Hz stimulation with an electrode decreased the primary and secondary generalized tonic–clonic seizures in the thalamic centromedian nucleus of patients with medically intractable epilepsy^49^. In patients with non-lesional temporal lobe epilepsy, HFS (130 Hz) prompted a reduction of interictal discharges and the absence of seizures^50^. In deep brain stimulation (DBS) with a high frequency electrical field, the stimulus inactivated the targeted neural structures. Recordings from the stimulated nucleus showed inhibition and/or decreased activity after the termination of the stimulus train^51,52^. Our results confirmed the inhibitory effects of high frequency stimulation. More importantly, we found that these frequency bands are equally powerful in inducing the post-stimulation, carry-over inhibition, suggesting that carry-over effects could be a general phenomenon under these clinically relevant stimulation frequencies.

Using a magnetic coil to generate high frequency pulses can be challenging when the coil is large. Generally, large coils possess significant energy-storage requirements and severe cooling issues. However, a few rTMS studies using large coils have provided evidence that high frequency magnetic stimulation is also inhibitory. For example, short duration, high frequency (20 Hz) rTMS trains have long-term anticonvulsant effects^53^. Animal work has also shown that, at high frequencies, rTMS could acutely decrease epileptic spike frequency^54^. We have also demonstrated that 20–400 Hz magnetic stimulation could inhibit epileptic-form activity in brain slices^40^. This study took advantage of the novel micromagnetic stimulation technology in generating high frequency pulses. Discoveries unveiling cellular behaviors under high frequency stimulation could propose clinically pertinent data for developing coil technology tailored to high frequency magnetic stimulation.

Third, NEURON modeling allows us to delineate the ion channel mechanisms underlying the carry-over effects. We found that high frequency magnetic stimulation motivated the fast oscillation of the membrane voltage (Fig. 7) and altered the ion channel dynamics that support the action potentials (Fig. 10). Specifically, the simulated voltage-clamp experiment indicated that, after magnetic stimulation, the sodium channel conductance decreased, while the potassium conductance increased (Fig. 11).

Finally, we found that methods designed to enhance the opening of sodium channels could prevent the carry-over effects. The first method targeted the application of enhanced membrane depolarization (Figs. 12 and 13) to activate the sodium channels to elicit action potentials. The second method granted the membrane a temporal “resting,” which led to de-inactivation of the sodium channels (Figs. 14 and 15) to increase sodium channel conductance.

It should be noted that, although the simulation work has captured many aspects of the experimental data, it cannot exclusively nor wholistically represent the complex biological reality. The modeling prediction about ion channel behavior under voltage-clamp (Fig. 11) should be tested experimentally, using two-electrode voltage-clamp recording (TEVC) technology since the size of *Aplysia* neuron is large and needs a sufficiently large current for the voltage-clamp experiment^55^. Aside from the mechanisms illuminated by this study, other cellular mechanisms could likewise contribute to the carry-over effects. Electric stimulation has been shown to substantially elevate levels of extracellular GABA, which suppresses neuron activity for several minutes^56^. Electromagnetic stimulation could also disrupt the homeostatic ionic microenvironment surrounding the neuron. To exemplify, electric stimulation could dramatically increase potassium ion concentration^57,58^, which would provide carry-over blockage effects to neurons.

### Implications to the control of carry-over effects in clinical neuromodulation with electric or magnetic stimulation

Regardless of the complicated mechanisms underlying high frequency magnetic stimulation and the associated carry-over effects, this work provides valuable insights to the control of carry-over effects in neuromodulation practices with electromagnetic stimulation.

First, this work highlights the importance of monitoring the dynamics of the brain that could contribute to carry-over effects in magnetic stimulation. Under physiological conditions, there are several factors that contribute to the fluctuation of the membrane potential, including channel noise, synaptic noise, uncertainty in spike timing, and background synaptic activity^59^. Specifically, somatic membrane potential (*V*_m_) fluctuations are driven by the convergence of synaptic inputs from diverse sources of upstream neurons; therefore, fluctuations in individual neuron activity could carry useful information regarding multidimensional population activity^60^. Monitoring the brain activity associated with these cellular biomarkers could be an important first step in the prediction and control of carry-over effects under different stimulation modalities, such as TMS and TDCS. Electroencephalography (EEG) is an ideal method to monitor the fluctuations in brain activity^61^. EEG can also be used to design closed-loop, purpose-driven stimuli to provide brain state guided stimulation^62,63^. In order to apply state-dependent brain stimulation to better deliver or control carry-over effects, real-time, multi-channel EEG data can be used to monitor the brain state online and modify stimulation parameters^64^.

Second, it is possible to control the carry-over effects in TMS by preconditioning the brain state with other stimulation modalities. It is essential to develop technology that can precondition the state of the neural network to enhance the stimulation outcome, including carry-over effects when they are needed for treatment purposes^65^. One prominent study in the motor domain demonstrated that high frequency rTMS (HF-rTMS) to the M1 area resulted in lasting depression of the motor evoked potential for up to 60 min post-stimulation, whereas LF-rTMS alone returned to baseline within 10 min^66^. Pre-conditioning the motor cortex with transcranial direct current stimulation (tDCS) followed by LF-rTMS can also elongate the carry-over effects of LF-rTMS on motor cortex excitability^67^.

Third, it is also reasonable to consider using pharmacological approaches to alter the excitability of the nervous system and control carry-over effects in rTMS practice. For example, HF-rTMS, combined with lorazepam, suppressed seizures in a rat kainate status epilepticus model^54^, with the combined method being more effective than rTMS alone. Similarly, when anticonvulsant phenytoin was administered to alter the network state, the magnetic field was more effective in decreasing audiogenic seizure severity in mice^68,69^. Future research should explore the possibility of improving complementary therapies by adjusting the excitability state of the nervous system, allowing improved control over carry-over effects.

Fourth, it is possible to design novel stimulation protocols that can directly alter the state of the network to control carry-over effects. This paper demonstrated that a “kick” current (Figs. 12 and 13) or a “resting” period (Figs. 14 and 15) in the stimulation protocol could cause a fluctuation in membrane voltage and the alteration of ion channel dynamics, thereby terminating the carry-over effects. While this data was derived from intracellular work with an injected depolarization/depolarization current, the “kick” current or “resting” phase could be easily achieved by extracellular stimulation with cathodic or anodic current, respectively^32^. For example, a recent study found that high frequency, orthodromic stimulation of the CA1 pyramidal neurons in the hippocampus could lead to neural inhibition in vivo. Interestingly, by inserting a single pulse to the high frequency stimulus train, neurons could be significantly activated^70^. At the network level, many protocols are available for perturbing the brain circuitry electrically or magnetically^13^, which could be utilized to terminate carry-over effects. On the other hand, when post-stimulation effects are desired and a persistent carry-over duration is expected for clinical purposes^71^, such network perturbation protocols should not be applied.

Lasting behavioral and physiological effects have been observed in various neuromodulation modalities with electric stimulation and in various brain regions, such as transcranial direct current stimulation (tDCS) of the motor cortex, transcranial alternating current stimulation (tACS) of the visual cortex^72^, and transcranial random noise stimulation (tRNS) of the parietal^73^. Our work in this paper provides compelling cellular insight to these clinical neuromodulation techniques and the fundamental mechanisms of delayed carry-over effects. However, since the cellular-level, in vitro experiments of this study used large invertebrate neurons, the parameters defining the magnetic field cannot be unerringly assimilated to the exact parameters used in clinical TMS on human subjects. There exists, unfortunately, a large gap between bench research and bed practice. It is, therefore, not our intention to evaluate our parameters in the context of clinical practice with TMS. The discussed clinical applications of this work primarily focus on the broad implications of the carry-over effects in magnetic stimulation and other neuromodulation modalities. Future work using human subjects, or mammalian animal models close to human subjects, under a large TMS coil with TMS protocols could provide deeper insights to the carry-over effects and, ultimately, the control of these effects in TMS practice.

## Methods

### In vitro* Aplysia* electrophysiology

The study was carried out in compliance with the ARRIVE guidelines. In total, 30 animals were used for the study. *Aplysia californica* (100–150 g) were obtained from Marinus Scientific (Newport Beach, CA) and were kept in artificial seawater at room temperature (20 ± 1 °C). Animals were anesthetized by an injection of isotonic MgCl_2_ (50% of body weight). The buccal ganglion was dissected and immersed in an *Aplysia* saline solution (pH 7.4), which contained 460 mM NaCl, 55 mM MgCl_2_.H_2_O, 11 mM CaCl_2_⋅2H_2_O, 10 mM KCl, and 10 mM Hepes.

For soma stimulation and recording using extracellular electrodes, the buccal ganglion was pinned down with the caudal side facing upwards. This allowed the visualization of the individual soma and closer positioning of the extracellular electrode to the target neuron. The extracellular electrodes were made by pulling single-barreled capillary glasses using a Flaming-Brown micropipette puller (P-30, Sutter Instrument). For extracellular recording from the soma, the size of the electrode tip was adjusted to be slightly smaller than the size of the cell bodies^32,38^. Electrodes made in the same pulling protocol were also used for nerve suction recording^74^ from buccal nerve II (BN2). Since the electrode tip was smaller than the diameter of the nerve, we broke the tip so that the nerve end could fit into the glass capillary. Extracellular recordings were amplified by a Model 1700 differential AC Amplifier (A-M Systems), which had a gain of 1000 and was filtered by a 1.0–500 Hz band pass filter.

For intracellular recording from buccal ganglion neurons, we removed the ganglion sheath to expose the cell bodies. The intracellular electrodes were made by pulling single-barreled capillary glasses using a Flaming-Brown micropipette puller (P-30, Sutter Instrument). The sharp electrode was back-filled with 3 M potassium acetate before use. For intracellular recording, the DC offset was eliminated, and the bridge was balanced before membrane penetration. Large jaw motor neurons on the caudal side of the buccal ganglion were recorded. These neurons have similar physiological properties and functions. To elicit action potentials, depolarization currents of various intensities were injected into the neuron. Intracellular signals were amplified using a DC-coupled amplifier (model 1600, A-M systems). To control the frequency of firing of recorded neurons, an isolated pulse stimulator (model 2100, A-M systems) was connected to the 1600 amplifier to deliver short pulses. Both intracellular and extracellular signals were digitized (25 kHz) by a CED 1401, recorded, and analyzed by Spike 2 software (version 7.2, Cambridge Electronic Design Limited).

### Miniature coil and high frequency magnetic stimulation

A magnetic generator was used for high frequency magnetic stimulation of ganglion neurons^40^. We used a commercially available inductor as a coil (MLG1005SR10JTD25, TDK U.S.A. Corporation, Uniondale, NY). The inductor was coated with acrylate copolymer enamel (Revlon, New York)^75^ for electric insulation and water impermeability of the exposed coil terminals during the electrophysiology experiments. An arbitrary function generator (AFG1022, Teletronix) produced the high frequency stimulation signal, which triggered large current pulses through a 1000 W power amplifier (Pyramid PB 717X 2 channel, Pyramid Car Audio, Brooklyn, NY, 11204) and drove the miniature coil. The amplifier was powered by a Triple Channel DC Power Supply (2231A-30-3, Keitheley).

For magnetic stimulation, the miniature coil was positioned by a micromanipulator above the buccal ganglion (Fig. 1). The coil was oriented so that its induced electric field was parallel to the ganglion buccal nerve II (BN2) axial to produce effective stimulation^76,77^ of the recorded motor neurons. To investigate the carry-over effects in the single buccal neurons, we applied a spectrum of stimulation frequencies to the coil (50–400 Hz). To estimate the waveform of the induced electric field, we measured close to the coil in the petri dish. Consistent with previous reports^26,78^, the miniature coil generated electric voltages in a biphasic shape^12^.

The impedance of the coil was measured at the beginning and end of each experiment to test its connectivity. Potential leakage of the coating coverage was also tested by measuring the impedance of the coil to the ground. If leakage current presents, an extremely large level of noise is generated. The local temperature around the coil was monitored with a thermocouple (HH11B, Omega Engineering, Norwalk, CT) to display the temperature with 0.1 °C resolution.

### Data analysis and statistics

Throughout the text, mean ± standard error (SE) was reported. All data was verified for normal distribution and homogeneity of variance by KS normality and Levene test, respectively. Statistical significance was determined with one-way repeated measures ANOVA, followed by multiple comparisons with Bonferroni test for data with equal variance and normal distribution, and Garnes-Howell test for data with no equal variance and/or no normal distribution using the Systat software (v. 13.1, Systat Software, Inc. San Jose, CA, USA). Effects were considered statistically significant at p < 0.05. We acquired multiple neurons from individual animals without subject averaging in the final statistics (nested design). Ethically, this method is favorable for reducing the number of animals in each experiment; however, this kind of data analysis, although commonly used in neuroscience, may reduce the statistical power^79^. To measure the frequency of spontaneous firing, the number of spikes was counted and subsequently divided by the time interval of interest. Duration of post-stimulation inhibition was measured between the end of the magnetic stimulation protocol and the start of neuronal firing.

### Computation of induced electric field in the vicinity of the neuron

To simulate the carry-over effects under magnetic stimulation at high frequency, a biophysics model of a circular coil was used to calculate the induced electric field around the cell^41^. Briefly, when an electric pulse was delivered to the coil, it generated a magnetic field around the coil (Fig. 2).

For a coil with a flowing current (I) inside, the magnetic field was calculated by

1 $$B={\mu }_{0}\frac{NI}{l}=\frac{{\mu }_{0}NV}{Rl}(1-{e}^{-\frac{tR}{L}})$$

for the rising phase of the pulse signal, and

2 $$B={\mu }_{0}\frac{NI}{l}=\frac{{\mu }_{0}NV}{Rl}{e}^{-\frac{tR}{L}}$$

for the falling phase of the pulse signal. Here, N was the loop of the coil, $l$ was the length of the coil, and *μ*_0_ = 4π × 10^−7^ H/m was the vacuum permeability. R was the resistance of the coil and V was the voltage measured across the coil.

Calculation of the induced electric field by a magnetic coil is based on Faraday’s law of induction. In the coordinate system in Fig. 2, after some calculation^41^, the induced electric field (outside the coil) during the rising phase was

3 $${E}_{x}=-\frac{V{\mu }_{0}N{{R}_{c}}^{2}}{2Ll}\frac{y}{{x}^{2}+{y}^{2}}{e}^{-\frac{tR}{L}}$$

4 $${E}_{y}=\frac{V{\mu }_{0}N{{R}_{c}}^{2}}{2Ll}\frac{x}{{x}^{2}+{y}^{2}}{e}^{-\frac{tR}{L}}$$

here, Rc is the radius of the coil. For the falling phase of the pulse,

5 $${E}_{x}=\frac{V{\mu }_{0}N{{R}_{c}}^{2}}{2Ll}\frac{y}{{x}^{2}+{y}^{2}}{e}^{-\frac{tR}{L}}$$

6 $${E}_{y}=-\frac{V{\mu }_{0}N{{R}_{c}}^{2}}{2Ll}\frac{x}{{x}^{2}+{y}^{2}}{e}^{-\frac{tR}{L}}$$

For the rising phase of the pulse, the electric potential distribution along the axon is

7 $$V(x)=\int {E}_{x}\left(x\right)dx=-\frac{V{\mu }_{0}N{{R}_{c}}^{2}}{2Ll}{\text{atan}}(\frac{x}{y}){e}^{-\frac{tR}{L}}$$

For the falling phase of the pulse, the electric potential distribution along the axon is

8 $$V(x)=\int {E}_{x}\left(x\right)dx=\frac{V{\mu }_{0}N{{R}_{c}}^{2}}{2Ll}{\text{atan}}(\frac{x}{y}){e}^{-\frac{tR}{L}}$$

We used the parameters of the miniature coil used in the experiments for these computational analyses (Table 1 in^6^).

### Multi-compartment NEURON modeling of an Aplysia ganglion neuron under magnetic stimulation

The carry-over effects were modeled with a multi-compartment soma-axon model of an *Aplysia* neuron^32^ using the NEURON simulation environment package^80^. Briefly, the modeled *Aplysia* neuron contained a spherical soma and a cylindrical axon (Fig. 2). Detailed geometrical parameters of the modeled neurons can be found elsewhere (Table 2 in^4^). The Hodgkin–Huxley (H–H)^45^ type of fast sodium, slow potassium, and leakage channels in the membrane were inserted into each compartment of the modeled neuron. Parameters that defined the biophysics properties of the H/H model can be found elsewhere (Table 3 in^4^).

During NEURON simulation, the electric voltage induced by the miniature coil (Eqs. 7 and 8) was applied to the modeled neuron using the “play” function^81^ in NEURON. The model was set to run at room temperature (20 °C), as in the electrophysiology experiments. We used biphasic, short pulses with alternating direction to represent the induced electric field. The duration of the pulse was 1 ms^41^. Both current-clamp and voltage-clamp experiments were performed. For V-clamp, the membrane was held from − 65 to 10 mV for 10 ms. Na and K currents were recorded under each voltage step. Duration of holding voltage was varied from 1 to 10 ms to reveal the sodium tail current.

## Data Availability

All data generated or analyzed during this study are included in this published article.
